# Functional diversification of two UGT80 enzymes required for steryl glucoside synthesis in *Arabidopsis*

**DOI:** 10.1093/jxb/eru410

**Published:** 2014-10-14

**Authors:** Daniel F. Stucky, James C. Arpin, Kathrin Schrick

**Affiliations:** ^1^Division of Biology, Kansas State University, Manhattan, KS 66506-4901, USA; ^2^Molecular, Cellular and Developmental Biology, Kansas State University, Manhattan, KS 66506-4901, USA; ^3^Department of Biochemistry and Molecular Biophysics, Kansas State University, Manhattan, KS 66506-4901, USA

**Keywords:** *Arabidopsis*, lipids, seed, steryl glycosides, UDP-glucose:sterol glucosyltransferase, UGT80A2, UGT80B1, UGT713B1.

## Abstract

Steryl glucosides (SG) are abundant steroid conjugates in plant membranes. Beyond structural roles in lipid bilayers, functions in sugar transport, storage, and/or signalling are predicted. UDP-glucose:sterol glucosyltransferase 80A2 (UGT80A2) and UGT80B1, which share similarity to fungal counterparts, are implicated in SG synthesis in *Arabidopsis thaliana*. A third related enzyme, which seems specific to the plant lineage, is encoded by *UGT713B1*/*At5g24750*. Genetic and biochemical approaches were employed to determine the role of each *UGT* gene in the production of specific SGs and acyl SGs (ASGs). Using direct infusion electrospray ionization tandem mass spectrometry (ESI-MS/MS), SG and acyl SG (ASG) contents of *ugt80* and *ugt713* mutants, and triple and double mutants were profiled in seeds. *In vitro* enzyme assays were performed to assay substrate preferences. Both *UGT80A2* and *UGT80B1*, but not *UGT713B1* were shown to be coordinately down-regulated during seed imbibition when SG levels decline, consistent with similar functions as UGT80 enzymes. *UGT80A2* was found to be required for normal levels of major SGs in seeds, whereas *UGT80B1* is involved in accumulation of minor SG and ASG compounds. Although the results demonstrate specific activities for UGT80A2 and UGT80B1, a role for UGT713B1 in SG synthesis was not supported. The data show that UGT80A2, the more highly conserved enzyme, is responsible for the bulk production of SGs in seeds, whereas UGT80B1 plays a critical accessory role. This study extends our knowledge of UGT80 enzymes and provides evidence for specialized functions for distinct classes of SG and ASG molecules in plants.

## Summary

Plants have two UDP-glc:sterol glucosyltransferase enzymes that synthesize sugar-conjugated forms of sterols known as steryl glucosides. We provide evidence for their specialized roles in the production of diverse sterol-derived compounds.

## Introduction

Plants synthesize a complex mixture of sterols comprised of sitosterol, campesterol, stigmasterol, and numerous minor constituents including brassicasterol and cholesterol (Benveniste, 2004). Sterol derivatives known as steryl glycosides (SG) and acyl steryl glycosides (ASG) are widespread among plants, yet little is known about their specific functions (Grille *et al.*, 2010). SGs are primarily found at the plasma membrane and have been identified in detergent resistant membrane preparations (Laloi *et al.*, 2007), suggesting structural functions within membrane microdomains. Aside from roles as membrane components, SGs may serve as sugar transporters or storage forms of sterols, and roles in signalling are also possible (Grille *et al.*, 2010).

SG is formed by the transfer of a sugar moiety, most commonly glucose, to the C3-hydroxyl of the sterol backbone, whereas ASG is formed in a subsequent step by acylation of the sugar with a fatty acid at the C6 position. Membrane-bound enzymes termed UDP-glucose:sterol glucosyltransferase 80 (UGT80) catalyse the reaction by which the sterol is glucosylated (Ury *et al.*, 1989). *Arabidopsis thaliana UGT80A2/At3g07020* and a related gene from *Avena sativa* (*UGT80A1*) were the first family members whose cDNA sequences were isolated from plants and whose corresponding enzymes were biochemically characterized (Warnecke and Heinz, 1994; Warnecke *et al.*, 1997). Sterol glucosyltransferase activity was verified *in vitro* for UGT80A2 with radiolabelled cholesterol and UDP-glucose as substrates (Warnecke *et al.*, 1997).

Based on mutant analysis in *Arabidopsis*, two genes coding for UGT80 enzymes, *UGT80A2* and *UGT80B1/At1g43620*, are implicated in SG production (DeBolt *et al.*, 2009). UGT80A2 and UGT80B1 proteins share ~51% identity and 62% similarity, although the enzyme activity of UGT80B1 has not been confirmed experimentally to date. *ugt80A2,B1* double mutants display a significant decrease in both SG and ASG levels in various plant tissues, consistent with redundant functions of the respective enzymes. The double mutants display multiple phenotypes in the seed including small embryo size, transparent testa, defects in flavonoid deposition, loss of the outer cuticle, and a reduction in aliphatic suberin and cutin-like polymers (DeBolt *et al.*, 2009). The mutants also display defects in the male gametophyte suggesting that SGs are critical for pollen coat formation and pollen viability (Choi *et al.*, 2014).

Although *ugt80A2,B1* seeds display significantly reduced SG production, they still maintain ~6–10% of the wild-type SG levels (Schrick *et al.*, 2012b). These residual SG levels in the *ugt80A2,B1* double mutant predict the existence of additional genes encoding enzymes able to catalyse sterol glycosylation. Sequence similarity searches using UGT80A2 and UGT80B1 uncovered a putative UGT enzyme encoded by At5g24750 (Grille *et al.*, 2010; Schrick *et al.*, 2012a), which we refer to as UGT713B1 in the present study. We utilized genetic analysis with an available T-DNA insertion mutant and enzyme assays to test the hypothesis that *UGT713B1*/*At5g14750* encodes a biologically active sterol glucosyltransferase enzyme.

Although the SG and ASG composition of *ugt80A2,B1* double mutants was previously characterized in seeds and other tissues (DeBolt *et al.*, 2009; Schrick *et al.*, 2012b), a detailed compositional analysis of each corresponding single mutant has not been reported to date. The main hypothesis that we test here is that UGT80A2 and UGT80B1 have distinct roles in SG synthesis in addition to redundant functions that were demonstrated previously (DeBolt *et al.*, 2009). In the present work we unveil striking differences in SG and ASG profiles between *ugt80A2* and *ugt80B1* mutants that hint at differences in substrate specificity of the respective enzymes. The possibility of overlapping as well as distinct sterol substrate preferences of UGT80A2 and UGT80B1 is especially relevant in light of the corresponding mutant phenotypes in the seed. Whereas *ugt80A2* mutants display only minor effects on growth, *ugt80B1* mutants, first described for their *transparent testa* phenotype (Focks *et al.*, 1999), seem to exhibit the seed phenotypes reported for *ugt80A2,B1* double mutants (DeBolt *et al.*, 2009). This study proposes functional differences between the *UGT80* enzymes from *Arabidopsis* and provides evidence for specialized roles for different classes of SG and ASG compounds.

## Materials and methods

### Plant material and growth conditions

The *Arabidopsis thaliana* Columbia (Col-0) accession was the genetic background used. T-DNA insertion alleles for *UGT80A2/At3g07020* (SALK_020939), *UGT80B1/At1g43620* (SALK_103581), and *UGT713B1*/*At5g24750* (SALK_080068) were from the Salk T-DNA collection (Alonso *et al.*, 2003). F_3_ lines segregating for a second insertion in *UGT713B1*/*At5g24750* (GABI_F21207) were from the GABI-Kat collection (Kleinboelting *et al.*, 2012). Crosses were performed with the homozygous Salk lines to construct each double mutant combination and the triple mutant. Genotyping was performed with primers listed in Supplementary Table S1. Plants were stratified at 4 °C for 3–5 d and grown on soil containing Metro-Mix 380, Vermiculite and Perlite (Hummert International, Topeka, KS) in a 7:3:2 ratio at 23 °C under continuous light. To determine seed masses, 100 seeds were counted and weighed for each genotype with 5 replicates each.

### Scanning electron microscopy

*Arabidopsis* seeds were sputter coated with a 60% gold–40% palladium alloy using the Desk II Sputter/Etch Unit (Denton Vacuum, LLC, Moorestown, NJ). Images were taken using a S-3500N instrument from Hitachi Science Systems, Ltd. (Ibaraki, Japan), equipped with a S-6542 Absorbed Electron Detector, C1005 SEM Cold/Hot Stage (Oxford Instruments Microanalysis Limited, Great Britain), and fitted with a Robinson backscattered detector (ETP-USA/Electron Detectors, Inc., Rocklin, CA). Images were processed using Adobe Photoshop CS4.

### Seed imbibition time-course

Seeds (~20mg dry weight) were imbibed on moist filter paper for 0, 1, 6, 12, or 24h and frozen in liquid nitrogen for RNA extraction. In parallel, the time-course samples destined for lipid extraction were immersed in 75 °C isopropanol with 0.01% BHT for 15min. Total lipids were extracted from dry and imbibed seeds as described below. For RNA isolation, seeds were ground in liquid nitrogen and extractions were done using the Ambion® RNAqueous Kit with Plant RNA Isolation Aid, followed by removal of genomic DNA using the Ambion® TURBO DNA-free^TM^ DNAse (Life Technologies, Carlsbad, CA). cDNA was synthesized with GoScript^TM^ Reverse Transcriptase (Promega North America, Madison, WI). Real-time PCR was performed with gene-specific primers listed in Supplementary Table S1 using iTaq Universal SYBR Green Supermix with the CFX96 Touch Real-Time PCR Detection System (Bio-Rad, Hercules, CA). *ACTIN-RELATED PROTEIN 6* (*ARP6*) served as the reference gene.

### Lipid extraction, electrospray ionization tandem mass spectrometry (ESI-MS/MS), and quadrupole time-of-flight (QTOF) mass spectrometry

Lipids were extracted from seeds as described previously (Schrick *et al.*, 2012b), a modification from (Bligh and Dyer, 1959). An internal standard mixture comprised of 0.5 nmol each of dilauroyl and diphytanoyl phosphatidylglycerol (PG(24:0) and PG(40:0); Avanti Polar Lipids, Alabaster, AL) was added to each sample before ESI-MS/MS analysis. Lipids were extracted from the yeast and *Escherichia coli* enzyme activity assays using a protocol similar to that described in Bligh and Dyer (1959). For the yeast samples, 0.25 nmol and for bacterial samples, 0.5 nmol of both PG(24:0) and PG(40:0) standards were added after and before lipid extraction, respectively. Unfractionated lipid extracts were introduced by continuous infusion into the ESI source on an API 4000 electrospray ionization (ESI) tandem mass spectrometer (Applied Biosystems, Foster City, CA). For the seed and yeast samples, the amount used for mass spectral analysis was 50 μl of 1ml lipid extract in 1.2ml solvent comprised of chloroform/methanol/300mM ammonium acetate in water (300/665/35), whereas for the bacterial samples the amount used was 5 μl of 500 μl total extract dissolved in 1.2ml of the solvent. To detect steryl glucuronides and steryl rhamnosides, 250 μl of the lipid extract dissolved in solvent was used. Samples were introduced by an autosampler (LC MiniPAL, CTC Analytics AG, Zwingen, Switzerland), fitted with an injection loop for the acquisition time, and presented to the ESI needle at 30 μl per min. Targeted methods were employed for analysis of internal standards, SG, and ASG lipid molecules. Internal standards were detected with a scan for neutral loss of the head group moiety, NL 189.04 (C_3_H_9_O_6_P + NH_3_) in the positive mode. For SG detection, a scan for neutral loss of the hexose moiety (glucose or galactose, NL 197.09, C_6_H_12_O_6_ + NH_3_) was used. ASG lipids were detected with neutral loss scans for the hexose moiety acylated with specified fatty acids (16:0, 18:3, 18:2, 18:1, 18:0, and 20:0) + NH_3_. Using cholesteryl β-d-glucuronide (Sigma-Aldrich, St. Louis, MO) as a standard, a scan was developed to detect glucuronic acid conjugates of sterols (steryl glucuronides). Supplementary Tables S2 and S3 list molecular weights, formulas, and neutral loss scans (in the positive mode) for each targeted lipid species. Routine polar lipids were detected as previously described (Xiao *et al.*, 2010). Sample runs, mass spectra detection, and data analysis were performed as described (Schrick *et al.*, 2012b). Sitosteryl rhamnoside, sitosteryl glucuronide, and sitosteryl glucoside were validated by accurate mass measurements as follows: The five wild-type replicates were pooled and products of the [M+NH_4_] positive ions were analysed by QTOF mass spectrometry (QSTAR Elite, Applied Biosystems) monitoring the appearance of the sitosterol fragment (*m/z*=397.3829). Sugars were identified as hexose, deoxyhexose, and hexuronic acid based on nominal masses, and specific assignments were based on previous findings indicating biological likelihood (Grille *et al.*, 2010).

### RNA extraction, cDNA synthesis, and RT-PCR

Seeds were surface sterilized and sown onto 0.8% (plant tissue culture grade) agar containing 0.5X MS (Murashige and Skoog, 1962) and 2% sucrose, stratified for 3–5 d at 4 ° C, and transferred to 23 °C and continuous light. RNA was isolated from 100mg of 7-day-old seedlings using RNeasy Plant Mini Kit and on-column RNase-Free DNase Set (Qiagen, Valencia, CA). RNA (2 μg) was used as a template for cDNA synthesis with GoScript Reverse Transcriptase (Promega North America, Madison, WI). Semi-quantitative RT-PCR reactions were performed using HotStarTaq (Qiagen, Valencia, CA) in a 25 μl volume according to the manufacturer’s protocol. RT-PCR products were resolved on a 1.5% agarose gel.

### DNA constructs

*UGT713B1/At5g24750* and *GCS*/At2g19880 cDNAs were amplified with Phusion High-Fidelity DNA Polymerase (Thermo Scientific USA) using the primers listed in Supplementary Table S1. Templates containing the *UGT80B1* and *GCS/At2g19880* cDNAs were provided by Seth DeBolt and Edgar Cahoon, respectively. The source of the Landsberg *erecta* (L*er*) *UGT713B1/At5g24750* was a cDNA library described in Grebe *et al.* (2000). The source of the Col-0 *UGT713B1/At5g24750* cDNA was GSLTFB67ZA04 (Genoscope, CNS, France). A pENTRTOPO clone for *UGT80A2* (G14575) was obtained from the ABRC stock center. *UGT80B1*, *UGT713B1/At5g24750*, and *GCS/At2g19880* cDNAs were cloned into pENTR^TM^-D-TOPO® (Life Technologies, Grand Island, NY) and entry vectors were moved into pYES-DEST52 or pDEST17 with Gateway® LR Clonase® II (Life Technologies). The Col-0 ecotype was used for all genes and the L*er* ecotype was cloned for *UGT713B1/At5g24750* in addition to Col-0. Sequences were confirmed using the gene-specific primers listed in Supplementary Table S1 and transformed into yeast (*Saccharomyces cerevisiae*) strain Y258 *ugt51* described below. Transformants were confirmed by sequencing the isolated plasmids.

### Yeast *UGT51* deletion

Yeast (*S. cerevisiae*) strain YLR189C (Thermo Scientific USA) contains a *KanMX* cassette replacing the native *UGT51* (*UGT51A1*) ORF. Genomic DNA was isolated from YLR189C and the *KanMX* cassette was amplified with primers flanking ~200bp 5ʹ and 3ʹ of the *UGT51* ORF: YLR189C_KanMX_F (5ʹ-CCCTGCTTACACCAGGGTTTATC-3ʹ) and YLR189C_KanMX_R (5ʹ-GCACAACATACGGTACGTTTAGG-3ʹ). The fragment was used to replace the *UGT51* ORF in Y258, a protein expression strain described in Li *et al.* (2010), by homologous recombination using standard LiAc transformation, followed by selection on G418 (Sigma-Aldrich, St. Louis, MO). Positive knockouts were confirmed by PCR products of expected sizes using the above-described flanking primers along with primers within the *KanMX* cassette: KanB (5ʹ-CTGCAGCGAGGAGCCGTAAT-3ʹ) and KanC (5ʹ-TGATTTTGATGACGAGCGTAAT-3ʹ).

### Enzyme assays

For the yeast assay, 200ml cultures containing the pYES-DEST52 vector with cDNA inserts encoding *UGT80A2*, *UGT80B1*, *UGT713B1*/*At5g24750*, or empty vector were grown at 30 °C to an OD_600_ of ~0.7 in synthetic –URA dropout mix (Sigma-Aldrich) with 20% raffinose. Protein expression was induced with addition of 3× yeast peptone media with 20% galactose. Cultures were incubated for 6h before harvesting cell pellets by centrifugation. Pellets were washed with distilled water, resuspended in lysis buffer (20mM Tris-HCl pH 7.9, 10mM MgCl_2_, 1mM EDTA, 5% (v/v) glycerol, 1mM DTT, 0.3M (NH_4_)_2_SO_4_) and lysed using a Mini BeadBeater (BioSpec Products, Bartlesville, OK) with 3×30 s cycles at max rpm. Lipid membrane fractions were separated by ultracentrifugation at 59 K rcf for 1.5h. The crude microsome pellet was resuspended in 0.5ml lysis buffer. Reaction mixtures consisted of 80 μl yeast lysate with 10 μl of either 4mM cholesterol (C8667, Sigma-Aldrich) or 4mM of a plant sterol mixture comprised of approximately 32% sitosterol, 46% stigmasterol, 13% campesterol, and 7% brassicasterol (2:1 mixture of a sitosterol extract from soybeans (48%; S5753) and stigmasterol (95%; S2424), Sigma-Aldrich). Two different substrates were used for each UGT80 protein: 360μM UDP-glucose (670120, Calbiochem) or UDP-glucuronic acid (U6751, Sigma-Aldrich). Each 100 μl reaction was incubated at 30 °C for 2h before quenching with 0.9ml 0.45% NaCl. A similar enzyme assay was conducted in *E. coli* strain BL21(DE3)pRARE with Col-0 *UGT80A2* and L*er UGT713B1*/*At5g24750* cDNA sequences expressed from pDEST17. The negative control was pDEST17 expressing a mutated glucosylceramide synthase GCS gene having a single nucleotide base deletion at codon 364, leading to a Ile to Thr change and a frameshift resulting in a truncated protein of 381 aa (~43kDa). Induction was with 1mM IPTG for 3h at 30 °C. Microsomal fractions were isolated by ultracentrifugation at 24K rpm for 1.5h. Enzyme reactions were performed with cholesterol and UDP-glucose as previously described (Warnecke *et al.*, 1997), except that the substrates were not radiolabelled.

### Accession numbers

**Fig. 1.**
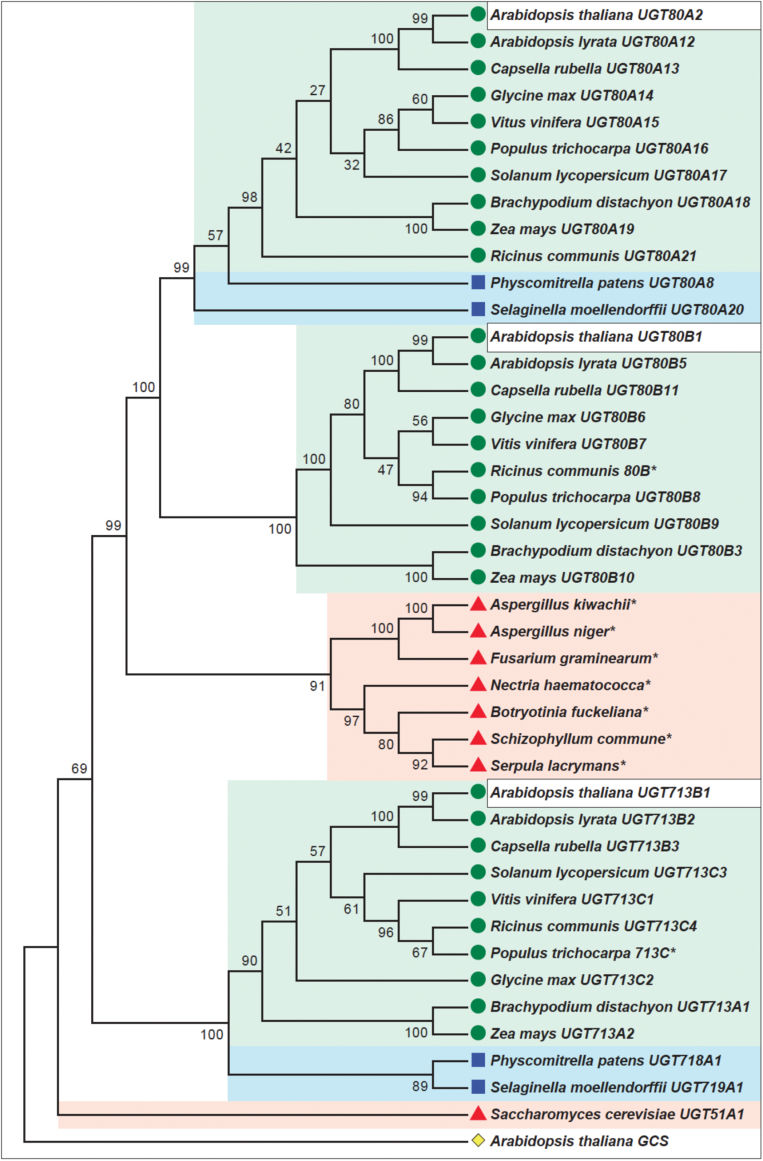
Neighbor-joining phylogenetic tree of UGT80- and UGT713-related proteins. *Arabidopsis thaliana* UGT80A2, UGT80B1, and UGT713B1 (white boxes) are conserved among vascular plants (green shading and circles) and mosses (blue shading and squares). UGT80 enzymes also share similarity with predicted proteins from various filamentous fungi (red shading and triangles), whereas UGT713 is limited to embryophytes. *Saccharomyces cerevisiae* UGT51A1, a functional sterol glucosyltransferase enzyme (red shading and triangle), was included for comparison. Asterisks denote sequences that were not assigned UGT names due to incomplete sequence or lack of EST support. The percentage of replicate trees in which the associated taxa clustered together in the bootstrap test (1000 replicates) is shown at the branches. Evolutionary distances were computed using the Poisson correction method and analysis was conducted in MEGA5.2.2 (Tamura *et al.*, 2011). The cladogram is rooted with respect to *Arabidopsis thaliana* At2g19880, a predicted glucosylceramide synthase (GCS) (yellow diamond).

Sequence data from this article can be found in the EMBL/GenBank data libraries under accession numbers NP_566297 (*UGT80A2*), NP_175027 (*UGT80B1*), NP_568452 (Col-0 *UGT713B1*), KJ396595 (Ler *UGT713B1*) and NM_127546 (*GCS*). Supplementary Table S4 lists the accession numbers for the protein sequences in Fig. 1. AGI locus identifiers: UGT80B1, At1g43620; UGT80A2, At3g07020; UGT713B1, At5g24750; GCS, At2g19880). The UGT nomenclature follows that previously defined by the UGT Nomenclature Committee (Mackenzie *et al.*, 1997).

## Results

### UGT713B1/At5g24750 is related to UGT80A2 and UGT80B1 and seems to be restricted to the plant lineage

We investigated the relatedness of the two previously identified UGT80 proteins to a third family member that was hypothesized to be involved in SG synthesis (Grille *et al.*, 2010; Schrick *et al.*, 2012a). Amino acid BLAST searches indicated only one other gene product encoded in the *Arabidopsis* genome that is closely related to *UGT80A2* and *UGT80B1*: the putative enzyme encoded by *At5g24750*, which was named UGT713B1 according to the standard UGT nomenclature (Mackenzie *et al.*, 1997). In comparison to UGT80A2 and UGT80B1 (637 and 615 amino acids, respectively), UGT713B1 is slightly smaller, coding for a 520 amino acid protein that has been annotated as a UDP-glycosyltransferase superfamily protein. Protein sequence alignments show ~38–39% similarity and ~21–23% identity with both UGT80A2 and UGT80B1.

Taking an unbiased approach, we searched for related UGT proteins in GenBank using NCBI BLAST. The evolutionary history of UGT80A2, UGT80B1, and UGT713B1 was then compared in a neighbor-joining phylogenetic tree that included UGT51A1, a functional sterol glucosyltransferase from the yeast *Saccharomyces cerevisiae* (Warnecke *et al.*, 1999) (Fig. 1; Supplementary Table S4). Although all three predicted proteins are conserved among embryophytes of the plant lineage, only UGT80A2 and UGT80B1, and not UGT713B1, were found to group with predicted fungal proteins (Fig. 1). This group of putative UGT proteins from seven different species of filamentous fungi, including several plant pathogens, have not yet been confirmed by EST support, and thus were not assigned UGT names here. The sequences were retained in our phylogenetic analysis, as they provide evidence for conserved function of the UGT80 clade.

Within the UGT80 branch of the cladogram, the moss sequences from *Physcomitrella patens* and *Selaginella moellendorffii*, named UGT80A8 and UGT80A20, respectively, are most closely aligned with UGT80A2 (Fig. 1). This observation suggests that UGT80A2 is the more conserved enzyme, whereas UGT80B1 and its close relatives seem to be restricted to the vascular plants. The UGT713 clade, although it does not include closely related enzymes from the fungal kingdom, is represented among species that span the embryophyte lineage. UGT713 proteins exhibit similarity to sequences from mosses that have been designated as UGT718 and UGT719 (Fig. 1).

### *UGT80A2* and *UGT80B1* but not *UGT713B1*/*At5g24750* transcript expression is correlated to SG levels in seeds

Gene expression patterns were surveyed for *UGT80A2*, *UGT80B1*, and *UGT713B1* using the publicly available transcriptome database Bio-Analytic Resource (BAR) *Arabidopsis* eFP browser. Notably, *UGT80A2* and *UGT80B1* seem to exhibit similar gene expression profiles in various tissues under numerous conditions. For dry seeds versus imbibed seeds, published microarray data indicate a decrease in both *UGT80A2* and *UGT80B1* gene expression, in contrast to an increase in *UGT713B1* transcript levels (Nakabayashi *et al.*, 2005). If UGT80A2 and UGT80B1 enzyme activities are reduced in imbibed seeds, SG levels would be expected to decline as a result.

**Fig. 2.**
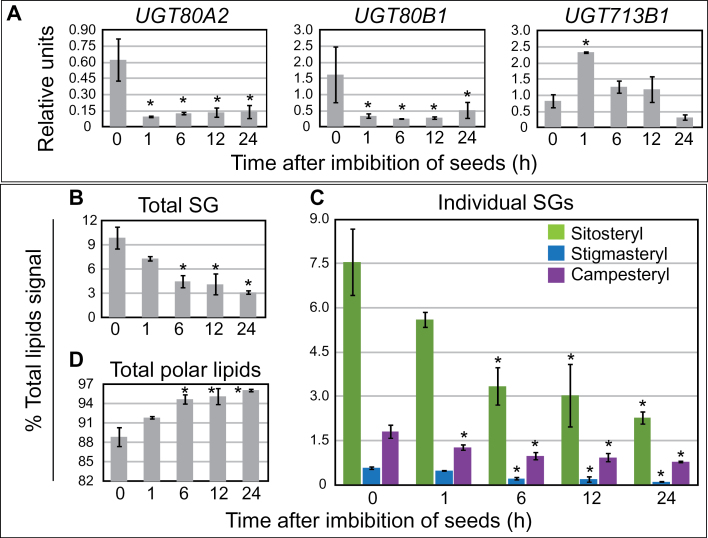
*UGT80* and *UGT713* mRNA expression in relation to SG levels in imbibed seeds. (A–D) Seeds were imbibed in water during a 24-hour time-course. Samples were taken at each time-point for RNA and lipid extractions in parallel experiments. Error bars denote standard deviations for *n*=3. Significant differences from the 0 hour time-point are indicated by an asterisk (two-tiered *t*-test, *P*≤0.05). (A) Quantitative real-time PCR was performed with cDNA synthesized from seed-derived RNA samples. Relative units normalized to the reference gene *ARP6* are plotted on the Y-axis. *UGT80A2* and *UGT80B1*, but not *UGT713B1* mRNA transcripts are down-regulated after one hour of imbibition. (B–D) SG and polar lipids are quantified as percent total signal by ESI-MS/MS. Total SG content (B), including sitosteryl, stigmasteryl, and campesteryl glucosides (C) decreases upon seed imbibition, whereas total polar lipids (D) increase upon seed imbibition.

We verified the published microarray data using quantitative real-time PCR (Fig. 2A) and monitored SG levels in seeds over a 24-hour time-course of imbibition. The real-time PCR data confirmed a ~5–6-fold decrease in both *UGT80A2* and *UGT80B1* transcript levels in imbibed seeds in comparison to dry seeds (Fig. 2A). In contrast, *UGT713B1* gene expression showed a ~3-fold increase within the first hour after imbibition. In parallel, seed samples from the 24-hour time-course were subjected to lipid analysis using direct infusion ESI-MS/MS. Consistent with a reduction in UGT80A2 and UGT80B1 activity we observed a significant decrease in total SG levels by the 6-hour time-point (Fig. 2B). The three major SGs (sitosteryl, stigmasteryl, and campesteryl) each showed similar reductions (Fig. 2C). In contrast, total polar lipids were found to increase significantly by the 6-hour time-point (Fig. 2D). This increase in polar lipids is likely to reflect an increase in metabolism upon seed imbibition. Therefore it is especially striking that during the time-course of imbibition, SG levels decrease. The finding that decreasing *UGT80A2* and *UGT80B1* gene expression correlates with reduced levels of SGs provides additional evidence for their function in SG synthesis. On the other hand, *UGT713B1* transcripts were not correlated with SG synthesis during seed imbibition.

### Characterization of *ugt80* and *ugt713* mutants and construction of the triple mutant

**Fig. 3.**
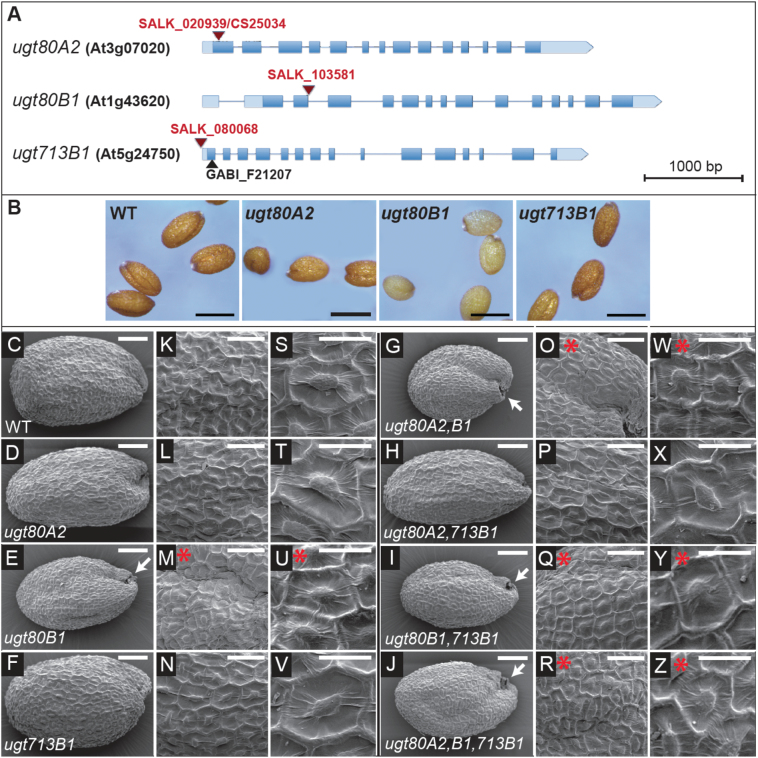
Description of *ugt80* and *ugt713* mutants and seed coat phenotypes. (A) Salk T-DNA insertion mutants for *UGT80A2*, *UGT80B1*, and *UGT713B1* are indicated in red and a GABI-Kat insertion mutant is shown in black. Exons are displayed as blue boxes. Light blue boxes indicate 3’- and 5’-UTRs. (B) Seed phenotypes for the *ugt80* mutants are shown. The *ugt80B1* seeds exhibit a transparent testa. Scale bars=500 μm. (C–Z) Scanning electron microscopy of dry seeds from each of eight genotypes: (C, K, S) Col-0 wild type (WT); (D, L, T) *ugt80A2*; (E, M, U) *ugt80B1*; (F, N, V) *ugt713B1*; (G, O, W) *ugt80A2,B1*; (H, P, X) *ugt80A2,713B1*; (I, Q, Y) *ugt80B1,713B1*; (J, R, Z) *ugt80A2,B1,713B1*. (C–F) Whole seeds. Scale bars=100 μm. (K–R) Magnification showing seed coat details. Scale bars=50 μm. (S–Z) Single mucilage secretory cells with columella in the centre. Scale bars=25 μm. Wild-type seeds have uniform seed coat cells with well-formed columella and cell walls. In contrast, *ugt80B1* mutants exhibit shallow columella (red asterisks) and a collapsed region near the funiculus (arrows). *ugt80A2* and *ugt713B1* mutants display no apparent differences in comparison to wild type.

We used genetic analysis to further probe the function of each individual *UGT80* gene in SG synthesis. Salk collection T-DNA insertion alleles of *ugt80A2*, *ugt80B1*, and *ugt713B1* were confirmed using the Columbia (Col-0) genetic background (Fig. 3A; Supplementary Fig. S1A). In addition we verified a second T-DNA insertion for *UGT713B1/At5g24750* from the GABI-Kat collection (GABI_F21207) whose phenotype seems to be indistinguishable from Col-0 wild type. Homozygous mutants for *ugt80B1* (SALK_103581) (at the exon-intron boundary following the second exon) and *ugt713B1* (SALK_080068) (in the 5’ UTR) were found to be mRNA knockdown alleles, whereas the homozygous mutant for *ugt80A2* (SALK_020939) (within the first exon), seems to express mRNA in semi-quantitative RT-PCR experiments (Fig. 3A; Supplementary Fig. S1B).

**Fig. 4.**
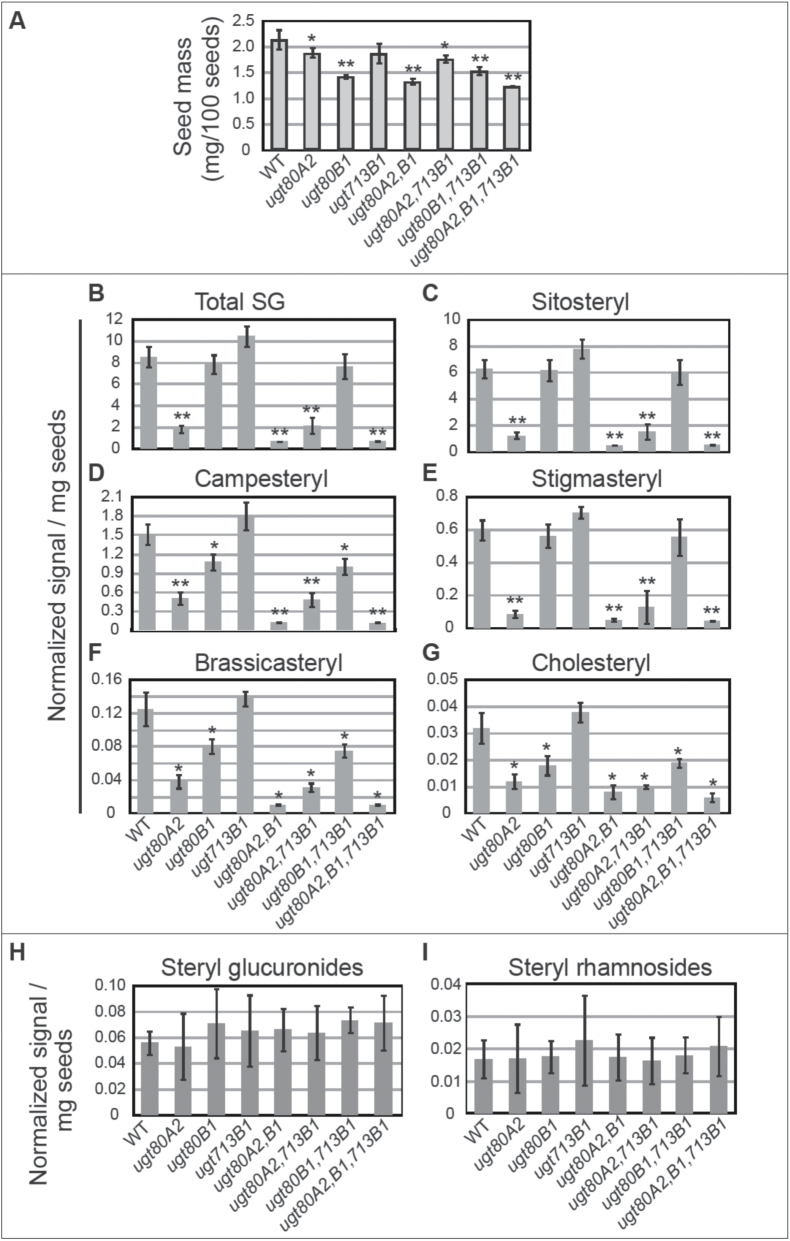
SG composition in wild-type and *ugt80* and *ugt713* mutant seeds. (A) Seed masses, corresponding to seeds subjected to lipid extraction and SG profiling are indicated for wild type (WT) and the *ugt80A2*, *ugt80B1*, *ugt713B1* single, double, and triple mutants. Significant differences from WT are seen for *ugt80A2* (two-tiered *t*-test, *P*≤0.05) (single asterisk) and *ugt80B1* mutants (*P*≤0.001) (double asterisk). (B) Total SG, and (C) sitosteryl, (D) campesteryl, (E) stigmasteryl, (F) brassicasteryl, and (G) cholesteryl glucoside levels were measured by ESI-MS/MS for the samples in (A). (H) Total steryl glucuronides and (I) total steryl rhamnosides are indicated for all the genotypes as in (B–G). Significant differences from WT are indicated by a single asterisk (two-tiered *t*-test, *P*≤0.05) or double asterisk (*P*≤0.0001). For (A–I), error bars denote standard deviations for *n*=5.

The Col-0 *ugt80B1* T-DNA insertion allele that we used in this study exhibits a transparent testa (*tt*) phenotype, like that from the Col-0 *tt15* (Focks *et al.*, 1999) and Wassilewskija (Ws-0) *ugt80B1* alleles (DeBolt *et al.*, 2009). In contrast, seed coloration from *ugt80A2* and *ugt713B1* mutants were indistinguishable from wild type (Fig. 3B). Crosses were made to construct each double mutant as well as the triple mutant. The seeds of all three double mutant combinations and the triple mutant exhibited no obvious phenotypes other than those associated with the *ugt80B1* mutation (Fig. 3E, G, I, J). Seed masses were compared for each of the genotypes, and only *ugt80B1* seeds displayed a striking reduction in mass, whereas *ugt80A2* mutants showed a small but significant decrease (Fig. 4A).

Seed coat morphologies for each mutant combination were observed by scanning electron microscopy (SEM) (Fig. 3C–Z). Although *ugt80A2* and *ugt713B1* mutant seeds showed no obvious differences in comparison to wild type, *ugt80B1* mutants were typically reduced in size (Fig. 3E, G, I, J). We also observed that the *ugt80B1* seeds displayed a collapsed region near the funiculus (Fig. 3E, G, I, J), and the mucilage secretory cells had shallow columella (Fig. 3U, W, Y, Z), consistent with previously reported data for *ugt80A2,B1* double mutant in the Ws-0 ecotype (DeBolt *et al.*, 2009). To investigate whether there is a compounded growth defect in the triple mutant, plants were monitored for 30 d on soil under uniform conditions (Supplementary Fig. S2). However, in comparison to wild type, no remarkable differences were seen in the growth or senescence for any of the mutant combinations.

### SG profiles in mutant seeds suggest specialized functions for UGT80A2 and UGT80B1, but no role in SG synthesis for UGT713B1/At5g24750

**Fig. 5.**
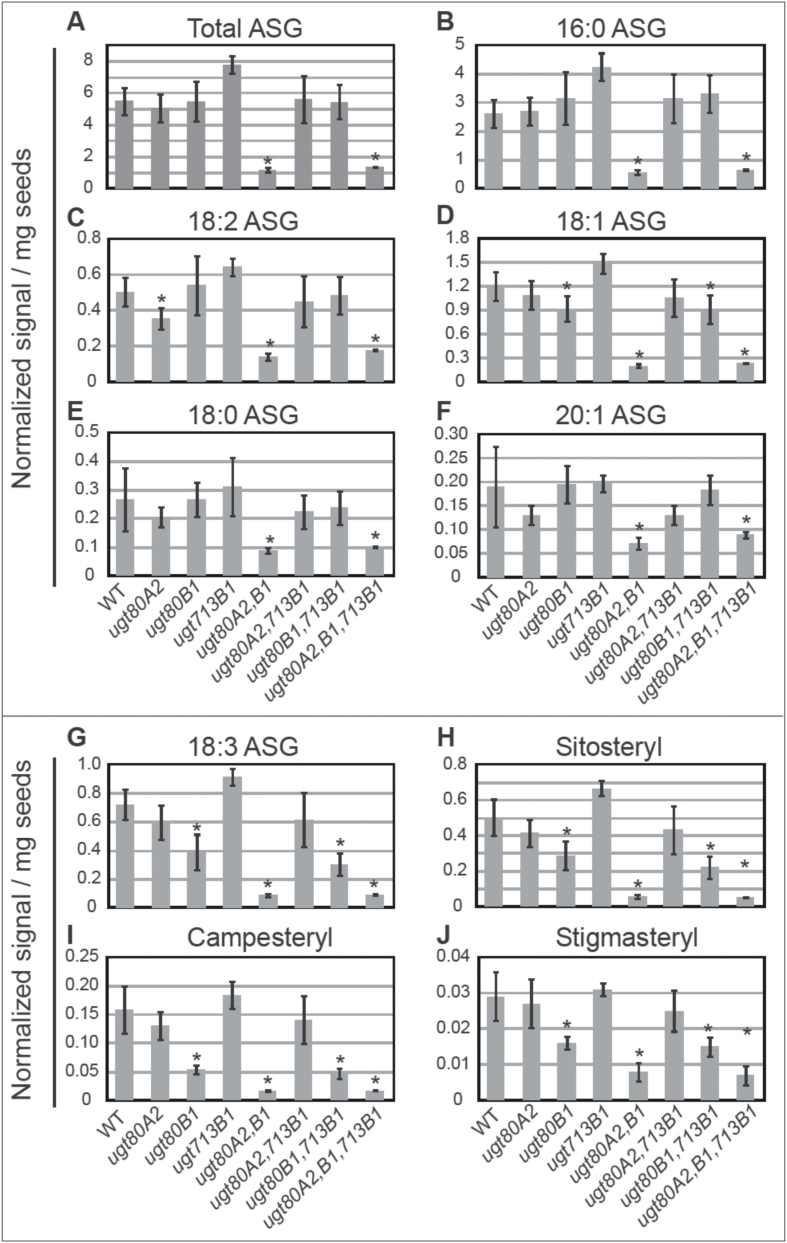
ASG composition in wild-type and *ugt80* and *ugt713* mutant seeds. For the samples in Fig. 4, ASG compounds were detected by ESI-MS/MS. (A) Total ASG, and (B) 16:0, (C) 18:2, (D) 18:1, (E) 18:0, and (F) 20:0 ASG are shown for wild type (WT), *ugt80A2*, *ugt80B1*, *ugt713B1*, each corresponding double mutant and the triple mutant. The *usg80A2,B1* double mutant shows significant decreases in ASG levels. For the single mutants the most striking reductions in ASG were observed in *ugt80B1* for 18:3 ASG: (G) total 18:3 ASGs, and (H) 18:3 sitosteryl, (I) 18:3 campesteryl, and (J) 18:3 stigmasteryl ASGs are indicated for all mutant combinations. 18:3 brassicasteryl and cholesteryl ASGs are given in Supplementary Table S5. Error bars denote standard deviations (*n*=5), and significant differences from WT are marked by an asterisk (two-tailed *t*-test, *P*≤0.05).

To examine the relationship between genotype, seed phenotype, and SG composition, we performed a targeted SG and ASG analysis for seeds from each *ugt* mutant combination in comparison to wild type using a direct infusion ESI-MS/MS approach (Figs 4B–G and 5; Supplementary Tables S2, S3, and S5). The results show that in Col-0 wild-type seeds, the most common SG is sitosteryl glucoside (~74%), followed by campesteryl (~18%), stigmasteryl (~7.0%), brassicasteryl (~1.5%), and cholesteryl (0.37%) glucosides, similar to the SG composition observed in wild-type seeds from the Ws-0 ecotype (Schrick *et al.*, 2012b). Previous reports indicate that the most common sugar moiety for SG is glucose, but others, such galactose, glucuronide, rhamnose, and xylose, are also reported (Grille *et al.*, 2010; Kovganko and Kashkan, 1999). Our analysis does not allow us to distinguish between the hexoses glucose and galactose from a mixture. As galactose is a very rare sugar moiety for SG in plants (Grille *et al.*, 2010), we assume that most of the hexose signal is from glucose. Specific scans for steryl glucuronides and steryl rhamnosides were also performed to detect these minor sterol derivatives (Fig. 4H, I). No differences between wild type and the mutants were observed, indicating that none of the corresponding *UGT* enzymes is responsible for the accumulation of steryl glucuronides or steryl rhamnosides in *Arabidopsis* seeds.

Among the single mutants, only *ugt80A2* seeds exhibited a significant decrease in total SG (~22% of the wild-type levels) (Fig. 4B; Supplementary Table S5). This phenotype in regard to SG accumulation indicates that the mRNA produced in *ugt80A2* mutants (Supplementary Fig. S1A) is either non-functional or reduced in activity. Among the different molecular species, *ugt80A2* mutants exhibited the greatest decreases in sitosteryl and stigmasteryl glucosides (~19 and 14% of wild-type levels, respectively), although reductions in campesteryl, brassicasteryl, and cholesteryl (~33, 30, and 38% of wild-type levels, respectively) glucosides were also detected (Fig. 4C–G). Surprisingly, *ugt80B1* mutants displayed a significant reduction only in campesteryl, brassicasteryl, and cholesteryl glucosides (~71, 65, and 56% of wild-type levels, respectively), but not sitosteryl or stigmasteryl glucosides (Fig. 4C–G). These data indicate that *UGT80A2* accounts for most of the sitosteryl and stigmasteryl glucoside production in seeds. In contrast to the *ugt80A2 or ugt80B1* mutants, *ugt713B1* single mutant seeds were not decreased in SG levels in comparison to wild type (Fig. 4B–G; Supplementary Table S5).

Among the double mutants, *ugt80A2,B1* seeds displayed the greatest reduction in total SG levels (~8.2% of wild-type levels) (Fig. 4B; Supplementary Table S5). The observed decrease is a significant reduction from the *ugt80A2* single mutant (~38% of *ugt80A2* levels, *P*≤0.005). Sitosteryl, stigmasteryl, campesteryl, and brassicasteryl glucosides were all reduced to ~8% of the wild-type level (~42, ~60, ~24, and ~26% of the *ugt80A2* level) (Fig. 4C–F), whereas the low levels of cholesteryl glucosides were reduced to ~25% of wild-type and ~67% of *ugt80A2* levels (Fig. 4G). These data are consistent with previous sterol profiles reported for *ugt80A2,B1* double mutant seeds in the Ws-0 accession (Schrick *et al.*, 2012b). The *ugt80A2,713B1* and *ugt80A2,B1* double mutants had similar sterol profiles to the *ugt80A2* and *ugt80B1* single mutants, respectively (Fig. 4B–G). The triple mutant profile showed a reduction in total SGs (~8.4% of wild type) very similar to the *ugt80A2,B1* double mutant (Fig. 4B–G). These data argue against the role of UGT713B1 in the synthesis of residual SGs in the *ugt80A2,B1* double mutant.

### ASG profiles indicate specific differences in *ugt80A2* versus *ugt80B1* mutants

As ASGs are synthesized from SGs, we expected decreases in these compounds in the *ugt80* mutants. However, none of the single mutants exhibited a decrease in total ASGs (Fig. 5A; Supplementary Table S5). We detected different species of acyl fatty acid modifications on SG in the seed samples, including palmitic (16:0), linolenic (18:3), linoleic (18:2), oleic (18:1), stearic (18:0), and eicosenoic (20:1) acids. In wild-type seeds, about half (~48%) of the ASGs detected were 16:0, followed by 18:1 (22%), 18:3 (~13%), 18:2 (~9.2%), 18:0 (~5.4%), and 20:1 (3.5%) (Fig. 5A–G; Supplementary Table S5). Among the different types of ASGs, we monitored sitosteryl, stigmasteryl, campesteryl, brassicasteryl, and campesteryl ASGs, and these reflected the observed SG compositions (Supplementary Table S5). In comparison to wild type, only *ugt80A2* seeds displayed a small but significant decrease in 18:2 ASGs (~70% of wild-type levels) (Fig. 5C). However, *ugt80B1* seeds, but not *ugt80A2* seeds, showed a significant decrease in 18:3 ASGs (~54% of the wild-type levels) (Fig. 5G–J), and to a lesser extent 18:1 ASGs (~77% of wild-type levels) (Fig. 5D). In contrast to *ugt80A2* and *ugt80B1*, the *ugt713B1* single mutant seeds were not significantly decreased in ASG levels in comparison to wild type (Fig. 5; Supplementary Table S5). The *ugt80A2,B1* double mutant showed the greatest decrease in total ASG (~21% of wild-type levels), whereas the triple mutant displayed a similar decrease (~24% of wild-type levels) (Fig. 5A). The *ugt80A2,B1* double mutant showed the following ASG decreases in comparison to wild type: 16:0 (~22%), 18:3 (~12%), 18:2 (~28%), 18:1 (~17%), 18:0 (33%), 20:1 (~38%) (Fig. 5B–G). As with the SG levels, the *ugt80A2,B1,713B1* triple mutant was not further decreased in SG levels, arguing against the postulate that *UGT713B1/At5g24750* accounts for residual levels detected in the double mutant. The significant decrease in ASG accumulation in the double mutant as compared with either single mutant further corroborates the functional redundancy of the UGT80A2 and UGT80B1 enzymes.

### Determination of UGT80A2 and UGT80B1 substrate use *in vitro*

**Fig. 6.**
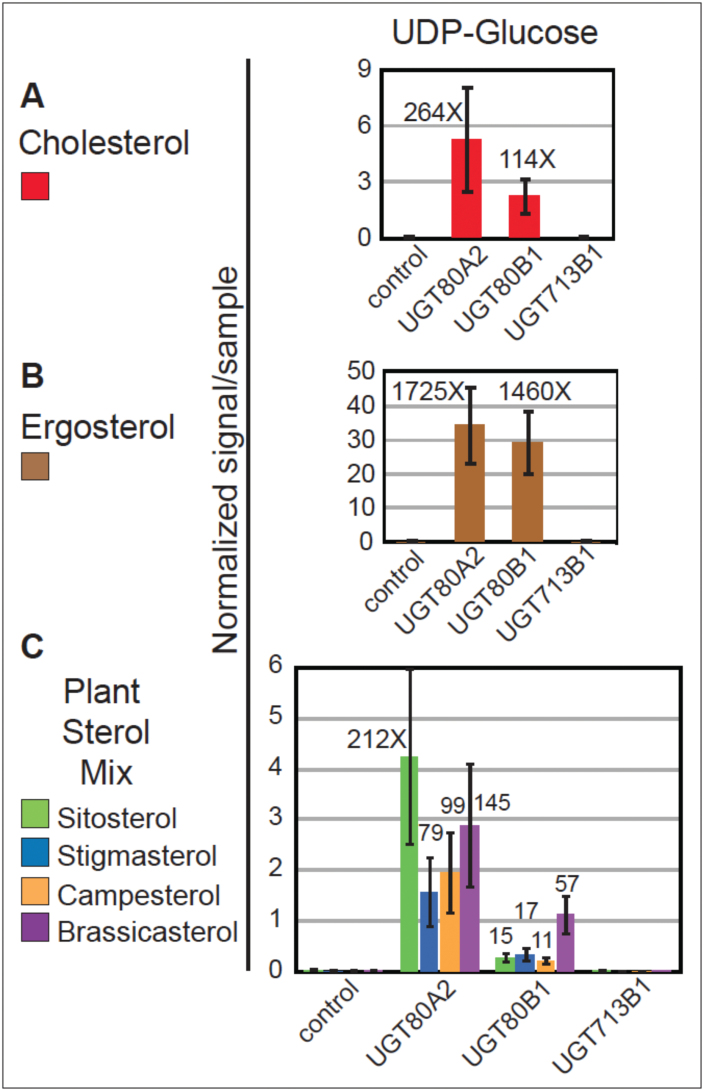
*In vitro* substrate specificity of UGT80A2 and UGT80B1 enzymes. (A–C) Yeast microsomes expressing the empty vector control, UGT80A2, UGT80B1, or UGT713B1 were incubated with sterol and UDP-glucose substrates. Reaction products were analysed by ESI-MS/MS and normalized signals per sample are shown. Error bars indicate standard deviations for *n*=3. Glucosylated sterols were detected for UGT80A2 and UGT80B1, but not for UGT713B1. Signals from the empty vector control ranged from 0.001 to 0.043 (Supplementary Table S6). Fold changes over the negative control were calculated using an average value of 0.020 for the control, and each fold-change represents a significant increase in activity over the control (*P*≤0.05). (A) UGT80A2 and UGT80B1 expression resulted in glucosylated cholesterol when using UDP-glucose as the substrate. (B) UGT80A2 and UGT80B1 displayed high activity with endogenous ergosterol for the UDP-glucose substrate. (C) UGT80A2 and UGT80B1 formed sitosteryl, stigmasteryl, campesteryl and brassicasteryl glucosides utilizing UDP-glucose in combination with a sterol mixture containing the corresponding sterols.

The UGT80A2, UGT80B1, and UGT713B1 enzymes were heterologously expressed in a yeast (*S. cerevisiae*) strain deleted for *UGT51/YLR189C*. Yeast *UGT51*, also known as *UGT51A1*, codes for an enzyme that glycosylates ergosterol (Warnecke *et al.*, 1999), and deletion thereof eliminates SG production. Yeast microsomal fractions individually expressing the *Arabidopsis* UGT80A2, UGT80B1, and UGT713B1 enzymes were incubated with various sterol substrates and UDP-glucose or UDP-glucuronide substrates to test for the production of SG. Subsequently, a direct infusion ESI-MS/MS approach was used to quantify SG products from the reactions (Schrick *et al.*, 2012b) (Fig. 6; Supplementary Tables S2, S3, and S6). The data indicate that both the UGT80A2 and UGT80B1 enzymes are able to glycosylate all sterols tested using UDP-glucose as a substrate. However, no detectable enzyme activity was observed with any of the sterol substrates with UDP-glucuronide as a substrate (Tables S3; S6).

With cholesterol and UDP-glucose as substrates, both UGT80A2 and UGT80B1 enzymes showed a greater than 100-fold increase in the production of SG over the control (Fig. 6A; Supplementary Table S6). Production of ergosterol glucosides was assayed in parallel (Fig. 6B; Supplementary Table S6). Although ergosterol was not added to the reactions, it was present, presumably as a sterol constituent of the yeast microsomes. Despite UGT80A2 and UGT80B1 being *Arabidopsis* proteins, the formation of ergosterol glucoside was detected at high levels in reactions containing UGT80A2 and UGT80B1 enzymes, consistent with their amino acid similarity to predicted fungal UGTs, as well as similarity to the yeast sterol glucosyltransferase enzyme UGT51A1 (Fig. 1). Both enzymes showed a greater than 1000-fold increase in activity in ergosteryl glucoside production using the UDP-glucose substrate in comparison to the control. It is likely that the activity of the UGT80 enzymes with ergosterol seems higher in comparison to the other sterol substrates analysed in our experiments owing to the endogenous abundance of ergosterol in yeast membranes.

Using the same yeast microsomal fractions, we also performed experiments in which a plant sterol mixture comprised of sitosterol, stigmasterol, campesterol, and brassicasterol was added to reactions containing the different UDP-substrates (Fig. 6C; Supplementary Table S6). In these experiments, both UGT80A2 and UGT80B1 enzymes displayed similar levels of activity with brassicasterol as the sterol substrate. However, the UGT80A2 enzyme produced significantly greater amounts of sitosteryl, stigmasteryl and campesteryl glucosides in comparison to UGT80B1. The results are consistent with possible substrate specificity from the SG profiling of wild-type and *ugt80* mutant seeds. Both UGT80A2 and UGT80B1 enzymes catalysed the production of the various SGs at a level that was significantly greater than the empty vector control. Therefore, these data further support the notion of functional redundancy for the two enzymes in plants in addition to the possibility of substrate preferences.

In contrast to UGT80A2 and UGT80B1, the UGT713B1 enzyme was unable to produce SGs with any of the sterol substrates used (Fig. 6; Supplementary Table S6). Expression of UGT80A2 and UGT713B1 in *E. coli* was consistent with the yeast assays with cholesterol (Supplementary Fig. S3; Fig. 6A). We used UGT80A2 as a positive control, as its glucosyltransferase activity in an *E. coli* expression system had been previously reported (Warnecke *et al.*, 1997). Enzyme activity was assayed by the relative amount of cholesterol glucoside produced using direct infusion ESI-MS/MS of lipid extracts. UGT80A2 displayed a greater than 100-fold increase in cholesteryl glucoside production compared with the negative control, whereas UGT713B1 displayed little or no activity. These data indicate that UGT713B1, in contrast to UGT80A2 and UGT80B1, lacks the ability to efficiently glucosylate sterols *in vitro* using either the yeast or *E. coli* expression systems.

## Discussion

### A role for UGT713B1/At5g24750 in SG synthesis is not supported

In this work we show that *ugt713B1* mutant seeds were not decreased in SG or ASG levels in comparison to wild type (Figs 4B–G and 5; Supplementary Table S5). Moreover, there was no further reduction of SG or ASG levels in double mutant combinations with *ugt80A2* or *ugt80B1*, or in the triple mutant. Therefore, UGT713B1/At5g24750 is not responsible for the residual production of SG in the *ugt80A2,B1* mutant seeds, as was initially hypothesized (Grille *et al.*, 2010). The absence of a mutant phenotype with respect to SG and ASG accumulation indicates that the UGT713B1 protein probably does not have, or has undetectable glycosylation activity on sterols in seeds. The inability to synthesize SGs *in vitro* (Fig. 6; Supplementary Table S6) further corroborates that *UGT713B1*/*At5g24750* does not encode a *bona fide* sterol glucosyltransferase enzyme as was previously hypothesized (Grille *et al.*, 2010; Schrick *et al.*, 2012b). This gene may have a unique substrate utilization and/or exhibit a novel enzymatic function that remains to be uncovered. From the phylogenetic analysis, the biological activity of UGT713B1 is predicted to be specific to plants including mosses (Fig. 1), and it may have diverged from the *UGT80* genes with the origin of the plant lineage. Other candidate genes required for SG and ASG biogenesis and metabolism in plant cells remain to be identified.

### Evidence for overlapping and distinct functions for UGT80A2 and UGT80B1 in SG synthesis

Functional redundancy is a recurrent theme in plant genomes. What does this mean at the level of enzyme activity? *UGT80A2* and *UGT80B1* are functionally redundant based on reduction of SG levels in the *ugt80A2,B1* double mutant, as was previously shown by DeBolt *et al.* (2009). However, before the present study, biochemical evidence for enzyme activity had only been reported for UGT80A2, using cholesterol and UDP-glucose as substrates (Warnecke *et al.*, 1997). Here we demonstrate that both the UGT80A2 and UGT80B1 enzymes from *Arabidopsis* display sterol glucosyltransferase activity *in vitro* (Fig. 6). Both enzymes can use UDP-glucose as a sugar substrate, and cholesterol or ergosterol as sterol substrates.

Intriguingly, we observed differences that suggest substrate preferences with respect to the major plant sterols (Fig. 6C). UGT80A2 seems to be the dominant enzyme, based on its ability to efficiently utilize sitosterol, campesterol, and stigmasterol as sterol substrates *in vitro*, and by the deficiencies seen in SG profiles from *ugt80A2* mutants (Fig. 4B–G; Supplementary Table S5). The data indicate that *UGT80A2* accounts for most of the sitosteryl and stigmasteryl glucoside production in seeds. Considering structural features, both sitosterol and stigmasterol are characterized by branching at the carbon 24 (C24) of the sterol side chain. Conversely, the *in vitro* assays suggest that UGT80B1 prefers brassicasterol, a sterol that lacks branching at the C24 of the side chain (Fig. 6C). Consistent with this, *ugt80B1* seeds exhibit reduced levels of brassicasteryl, cholesteryl, and campesteryl SGs, all three of which represent sterols with unbranched side chains (Fig. 4B–G; Supplementary Table S5). These observations point to a structural basis underlying the suggested substrate preference.

The observed differences between *Arabidopsis* UGT80A2 and UGT80B1 are also intriguing in light of the phylogenetic tree data (Fig. 1). Although both enzymes are related to fungal counterparts, UGT80A2, unlike UGT80B1, also has homologues among the mosses, suggesting that it represents the more ancient enzyme. UGT80B1, on the other hand, may have arisen in the vascular plants to play a more specialized role in SG synthesis, as described below.

### Mutant phenotypes indicate that UGT80B1 plays a specialized role in SG synthesis

The conclusion that UGT80A2 is the major UGT80 enzyme for SG production is especially surprising considering that *ugt80B1* seeds and not *ugt80A2* seeds display a visible phenotype, as observed by the transparent testa and abnormal morphology of the mucilage secretory cells of the seed coat (DeBolt et al, 2009; Fig. 3). One possibility, as described above, is that the observed *ugt80B1* phenotypes are a consequence of the differential substrate specificity between the two enzymes. Alternatively, or in addition, UGT80B1 synthesizes SGs that are more specifically localized, either in specific cell-types or in specific subcellular domains and were therefore missed in our SG profiling, which detects the total amount of lipid from whole seeds. In support of this idea, the ASG profiling data indicated that *ugt80B1* mutants have significantly reduced levels of sitosteryl, stigmasteryl, and campesteryl 18:3 ASGs (Fig. 5G–J). This observation is surprising given the SG profiling data that shows significant reduction of sitosteryl and stigmasterol glucosides in *ugt80A2*, but not in *ugt80B1* mutants (Fig. 4B–G). Taken together, the data suggest that in addition to possible substrate preference differences, localized expression of UGT80 enzymes and acylating enzymes contribute to the pools of diverse SG and ASG compounds in plants.

Based on the phenotype of *ugt80A2,B1* mutants, one recent study has implicated SGs in pollen viability (Choi *et al.*, 2014). However a clear mechanistic understanding of the functions of SGs and ASGs in plant cells is lacking. One postulate put forth more than ten years ago was that SGs could act as primers for cellulose synthesis (Peng *et al.*, 2002). It is also possible that SGs might serve as primers for other types of polymers that contain glucose or other sugars. Additional information about the UGT80 enzymes will be crucial in making associations to novel phenotypes of *ugt80* mutants as they are uncovered.

It is surprising that seeds from *ugt80B1* single mutants exhibit most if not all of the phenotypes as the *ugt80A2,B1* double mutant, despite significant differences in SG and ASG levels (Figs 3–5). These results suggest that a deficiency in minor compounds, such as 18:3 or 18:1 ASG, or SGs that are significantly reduced in *ugt80B1* such as cholesteryl, campesteryl, and/or brassicasteryl glucosides, may be responsible for the observed defects in the seed. The findings predict novel functions for distinct classes of SG and ASG molecules in seeds and in other plant tissues. Alternatively, UGT80B1 may catalyse the production of other sterol derivatives that are not synthesized by UGT80A2 and remain to be identified. The localized activity of each enzyme is likely to be critical for function. It is intriguing that *UGT80A2* and *UGT80B1* are co-transcriptionally regulated, for example, during seed imbibition (Fig. 2), suggesting that they act in concert, each playing a distinct role in SG synthesis in plant cells. Future studies that address the subcellular localization of these enzymes, including their closest relative encoded by the *UGT713B1/At5g24750*, may shed additional light on their individual functions in plants.

## Supplementary data

Supplementary data are available at *JXB* online

Fig. S1. Molecular characterization of T-DNA insertion mutants for *UGT80A2*, *UGT80B1*, and *UGT713B1*/*At5g24750* in the Col-0 accession.

Fig. S2. Plant growth comparison.

Fig. S3. Expression of UGT80A2 and UGT713B1/At5g24750 enzymes in *E. coli*.

Table S1. Oligonucleotides used in this study.

Table S2. Mass spectral scan values for SGs and ASGs.

Table S3. Mass spectral scan values for steryl glucuronides and rhamnosides.

Table S4. List of protein sequences for phylogenetic tree from Fig. 1.

Table S5. SG, ASG, steryl glucuronides, and steryl rhamnosides quantification in *ugt80* and *ugt713* mutant seeds.

Table S6. *In vitro* steryl glucosyltransferase activity of UGT80A2, UGT80B1, and UGT713B1 enzymes.

Supplementary Data
