# Supplementary material for: Functional diversification of two UGT80 enzymes required for steryl glucoside synthesis in Arabidopsis
Source: J Exp Bot. 2014 Oct 14;66(1):189–201. doi: 10.1093/jxb/eru410 (PMC4265157; doi:10.1093/jxb/eru410)
Supplement: Supplementary Data [file supp_66_1_189__index.html]

Functional diversification of two UGT80 enzymes required for steryl glucoside synthesis in Arabidopsis — Functional diversification of two UGT80 enzymes required for steryl glucoside synthesis in Arabidopsis — Functional diversification of two UGT80 enzymes required for steryl glucoside synthesis in Arabidopsis — Supplementary Data 

# Functional diversification of two UGT80 enzymes required for steryl glucoside synthesis in *Arabidopsis*

## Supplementary Data

Data files

**Files in this Data Supplement:**

- Supplementary Data - Supplementary Data
