# Supplementary material for: Functional diversification of two UGT80 enzymes required for steryl glucoside synthesis in Arabidopsis
Source: J Exp Bot. 2014 Oct 14;66(1):189–201. doi: 10.1093/jxb/eru410 (PMC4265157; doi:10.1093/jxb/eru410)
Supplement: Supplementary Data [file supp_eru410_jexbot130930_file001.pdf]

Functional diversification of two UGT80 enzymes required for steryl glucoside synthesis in *Arabidopsis* seeds

Daniel F. Stucky, James C. Arpin, and Kathrin Schrick

Supplementary Data

Fig. S1. Molecular characterization of T-DNA insertion mutants for *UGT80A2*, *UGT80B1* and *UGT713B1*/*At5g24750* in the Col-0 accession.

Fig. S2. Plant growth comparison.

Fig. S3. Expression of UGT80A2 and UGT713B1/At5g24750 enzymes in *E. coli*.

Table S1. Oligonucleotides used in this study.

Table S2. Mass spectral scan values for SGs and ASGs.

Table S3. Mass spectral scan values for steryl glucuronides and rhamnosides.

Table S4. List of protein sequences for phylogenetic tree from Fig. 1.

Table S5. SG, ASG, steryl glucuronides, and steryl rhamnosides quantification in *ugt80* and *ugt713* mutant seeds.

Table S6. *In vitro* steryl glucosyltransferase activity of UGT80A2, UGT80B1, and UGT713B1 enyzmes.

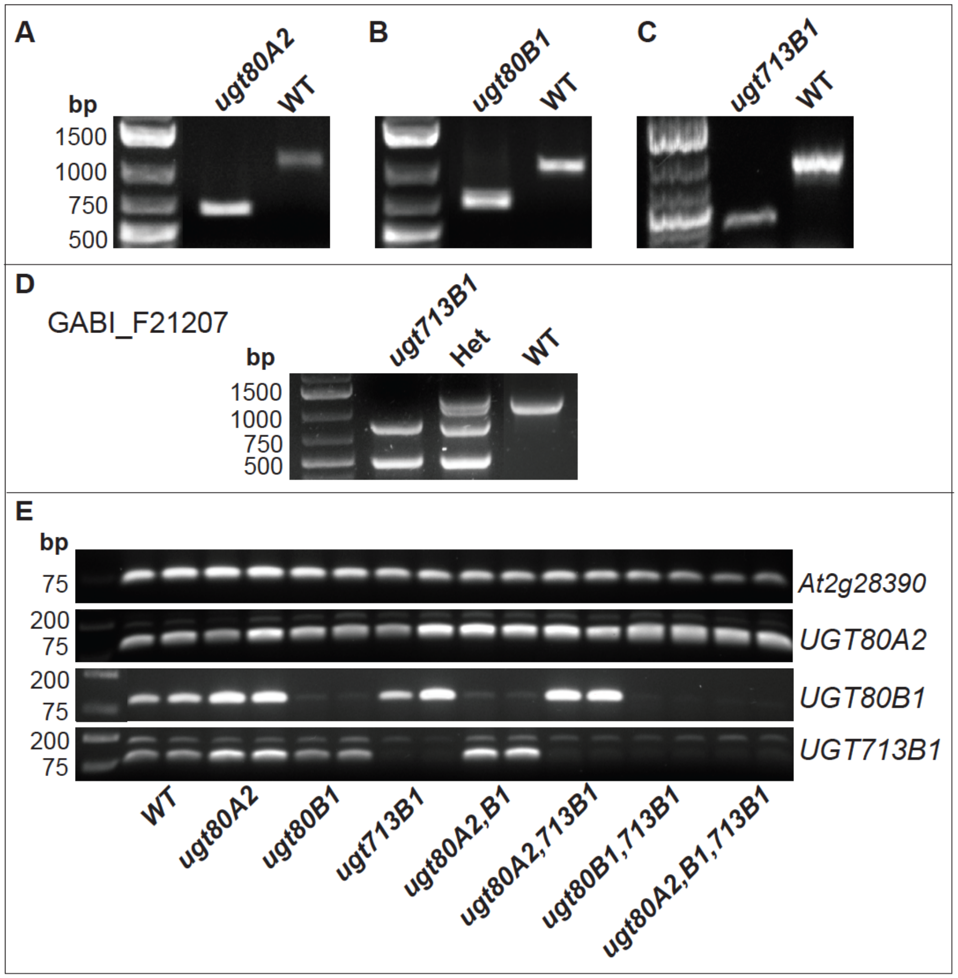

Figure S1. Molecular characterization of T-DNA insertion mutants for *UGT80A2*, *UGT80B1* and *UGT713B1/At5g24750* in the Col-0 accession.

(A-C) PCR genotyping is shown for the SALK T-DNA insertion lines. In mutant alleles a ~0.5-0.8 kb fragment is amplified versus a larger ~1.0 kb fragment in wild-type (WT).

(D) PCR genotyping for the GABI-Kat T-DNA insertion line corresponding to *UGT713B1*. For the mutant allele, two fragments are amplified while a larger ~1.0 kb fragment is observed in WT. Heterozygous plants express the combination of fragments.

(E) RNA was isolated from the *ugt80A2*, *ugt80B1* and *ugt713B1* single, double and the triple mutant seedlings, and semi-quantitative RT-PCR was performed with gene-specific primers for the three *UGT* genes. The SALK T-DNA insertion lines shown in (A) were used for the analysis. Two independent biological replicates are shown for each genotype. Transcript expression of Sand family member At2g28390 served as the reference gene. Both *ugt80B1* and *ugt713B1* mutant alleles represent mRNA knock-downs.

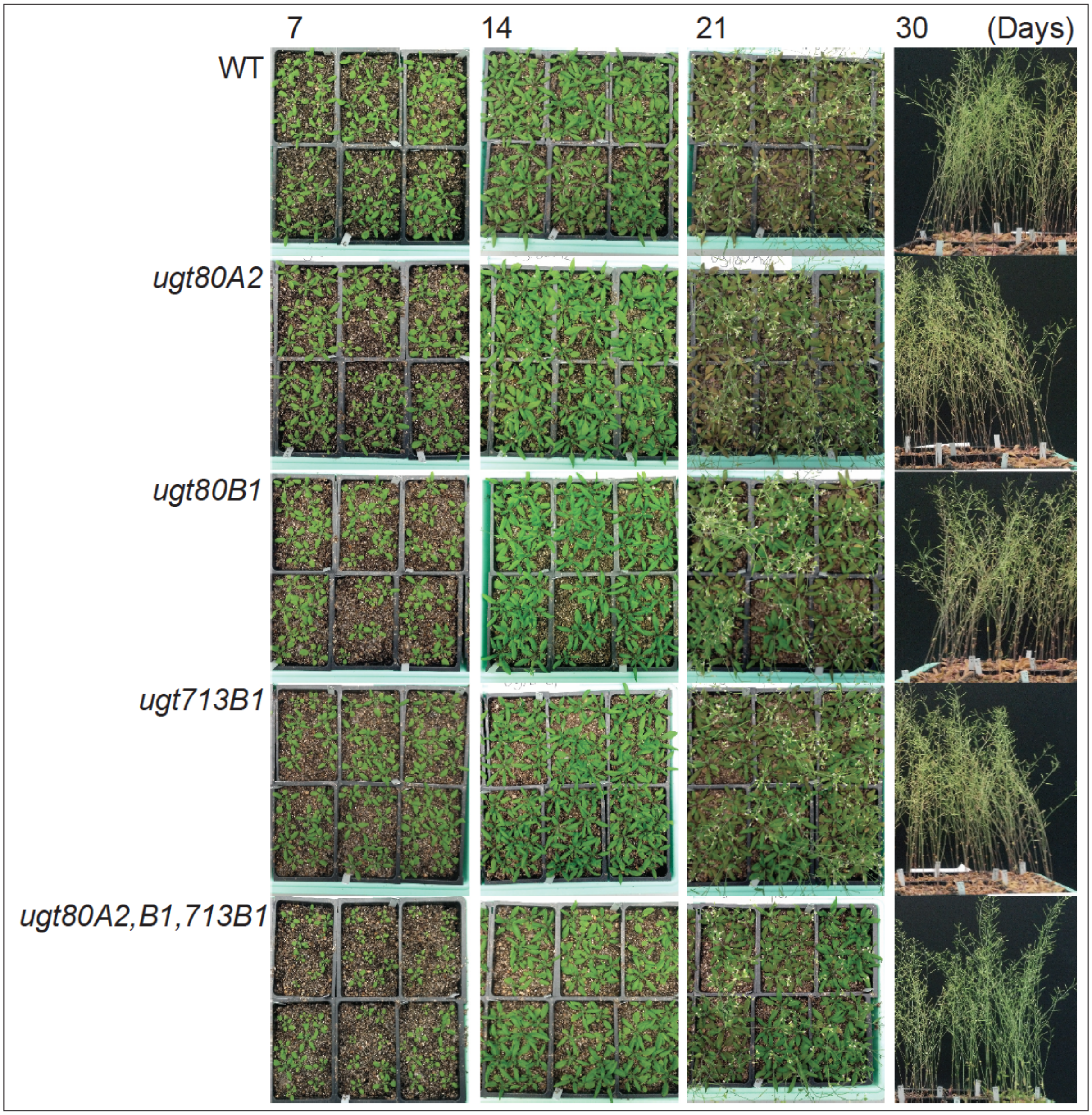

Figure S2. Plant growth comparison. *Arabidopsis* Col wild-type (WT), *ugt80A2, ugt80B1,* and *ugt713B1* mutants and the triple mutant were grown under continuous light for 30 days and monitored for differences in growth and senescence. Water (500 ml per tray of 12 pots) was given every two days. No obvious defects among the mutants were observed in comparison to WT.

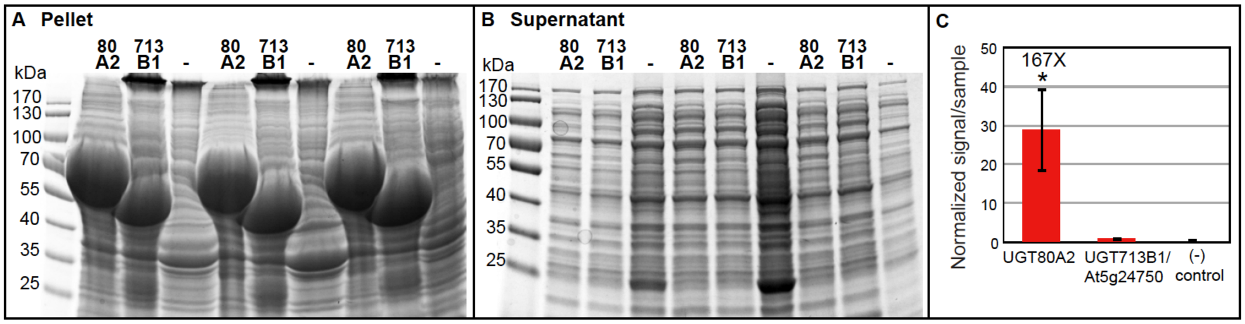

Figure S3. Expression of UGT80A2 and UGT713B1/At5g24750 enzymes in *E. coli.* Microsomal fractions were prepared from *E. coli* cultures and used for *in vitro* enzyme activity assays.

(A-B) Protein expression was analyzed by SDS-PAGE. Three biological replicates for UGT80A2 (80A2), UGT713B1 (713B1) and the negative control (-) are shown. The negative control consisted of a truncated GCS/At2g19880 gene.

(A) Pellet fractions indicate the presence of ~69 and ~59 kDa proteins for samples containing UGT80A2 and UGT713B1, respectively.

(B) Supernatants lack the expressed proteins.

(C) Pellet fractions expressing UGT enzymes or the negative control (-) were incubated with cholesterol and UDP-glucose. Lipid extractions were analyzed by ESI-MS/MS and normalized signals per sample are shown. Enzyme activity for production of cholesteryl glucoside was observed for UGT80A2 whereas little or no activity was detected for UGT713B1. Fold change over the control is indicated for UGT80A2. Error bars denote standard deviations (n=3). A significant increase in cholesteryl glucoside production in comparison to the control is marked by an asterisk (Two-tiered *t*-test, $P \leq 0.05$).

Table S1. Oligonucleotides used in this study.

**A. Primers genotyping *ugt80* and *ugt713* mutant alleles.**	
**Name**	**5'-3' sequence**
ugt80A2_SALK_020939_R	CTT AGC AGC ATC CTC AGC AAG
ugt80A2_ SALK_020939_L	TGG AGA CCT TTT GCG TGT AAG
ugt80B1_ SALK_103581_R	ATT GTG TGG GTT TAG GCA GTG
ugt80B1_ SALK_103581_L	TGG GTA GAG CCA TTT CAT TTG
ugt713B1_ SALK_080068_R	TGG TTT GTT GTG AAG TGA TCG
ugt713B1_ SALK_080068_L	TTC CGA AAA TGG TTC TGA ATG
ugt713B1_GABI_212F07_LP_1150	TTC CGA AAA TGG TTC TGA ATG
ugt713B1_GABI_212F07_RP_1150	AAG GAA AAT GTT TGC GTT GTG
LBb1 (for *ugt80A2* and *ugt80B1*)	GCG TGG ACC GCT TGC TGC AAC T
LBb1.3 (for *ugt713B1*)	ATT TTG CCG ATT TCG GAA C
o8409 GABI-KAT LB	ATA TTG ACC ATC ATA CTC ATT GC
**B. Primers for real-time PCR and semi-quantitative RT-PCR.** Amplicon sizes are indicated in parentheses adjacent to forward primer.	
UGT80A2_37_F (80 bp)	CGCATACGCAAATTCGAGATACACG
UGT80A2_116_R	GCATCGTGATTCTCTCTATCTCCACC
UGT80A2_2712_F (103 bp)	TGGAGTCCTCACCTTGTACCAAAGC
UGT80A2_2896_R	CTGCAGGAGGTTCATAGTTGGATGC
UGT80B1_563_F (104 bp)	CTGATGGGCATAGGGGACTAGACCA
UGT80B1_666_R	TGTCAAGGACCGAGAGTACTGAATCTCA
UGT713B1_245_F (87 bp)	TTCCTATCAATTCTCCTCCTGCTCTGTC
UGT713B1_331_R	CCTCCAAAAACATTTTCCTCAGCG
At2g28390_F (61 bp)	AACTCTATGCAGCATTTGATCCACT (Czechowski et al., 2005)
At2g28390_R	TGATTGCATATCTTTATCGCCATC (Czechowski et al., 2005)
ARP6_real-time_F (130 bp)	TAACAACTCAGGAGGACCCCA (Dekkers et al., 2012)
ARP6_real-time_R	CTACGACACCGAGCTGAT (Dekkers et al., 2012)

Czechowski T, Stitt M, Altmann T, Udvardi MK, Scheible WR (2005) Genome-wide identification and testing of superior reference genes for transcript normalization in *Arabidopsis*. Plant Physiol. **139:** 5-17

Dekkers, BJW, Willems, L, Bassel, GW, van Bolderen-Veldkamp, RPM, Ligterink, W, Hilhorst, HWM, Bentsink, L. (2012). Identification of reference genes for RT-qPCR expression analysis in *Arabidopsis* and tomato seeds. Plant & Cell Physiol. **53:** 28-37

Table S2. Mass spectral scan values for SGs and ASGs.

Class	Formula [M]	Scan mode	Mass of intact ion analyzed $[M+NH_4]^+$ (*m/z*)
**Steryl Glucoside (SG)**			
cholesteryl	$C_{33}H_{56}O_6$	+NL 197	566.4
ergosteryl	$C_{34}H_{54}O_6$	+NL 197	576.4
brassicasteryl	$C_{34}H_{56}O_6$	+NL 197	578.4
campesteryl	$C_{34}H_{58}O_6$	+NL 197	580.4
stigmasteryl	$C_{35}H_{58}O_6$	+NL 197	592.4
sitosteryl	$C_{35}H_{60}O_6$	+NL 197	594.4
**Acyl Steryl Glucoside (ASG)**			
16:0 cholesteryl	$C_{49}H_{90}O_7$	+NL 435	804.7
16:0 brassicasteryl	$C_{50}H_{90}O_7$	+NL 435	816.7
16:0 campesteryl	$C_{50}H_{92}O_7$	+NL 435	818.7
16:0 stigmasteryl	$C_{51}H_{92}O_7$	+NL 435	830.7
16:0 sitosteryl	$C_{51}H_{94}O_7$	+NL 435	832.7
18:3 cholesteryl	$C_{51}H_{88}O_7$	+NL 457	826.7
18:3 brassicasteryl	$C_{52}H_{88}O_7$	+NL 457	838.7
18:3 campesteryl	$C_{52}H_{90}O_7$	+NL 457	840.7
18:3 stigmasteryl	$C_{53}H_{90}O_7$	+NL 457	852.7
18:3 sitosteryl	$C_{53}H_{92}O_7$	+NL 457	854.7
18:2 cholesteryl	$C_{51}H_{90}O_7$	+NL 459	828.7
18:2 brassicasteryl	$C_{52}H_{90}O_7$	+NL 459	840.7
18:2 campesteryl	$C_{52}H_{92}O_7$	+NL 459	842.7
18:2 stigmasteryl	$C_{53}H_{92}O_7$	+NL 459	854.7
18:2 sitosteryl	$C_{53}H_{94}O_7$	+NL 459	856.7
18:1 cholesteryl	$C_{51}H_{92}O_7$	+NL 461	830.7
18:1 brassicasteryl	$C_{52}H_{92}O_7$	+NL 461	842.7
18:1 campesteryl	$C_{52}H_{94}O_7$	+NL 461	844.7
18:1 stigmasteryl	$C_{53}H_{94}O_7$	+NL 461	856.7
18:1 sitosteryl	$C_{53}H_{96}O_7$	+NL 461	858.7
18:0 cholesteryl	$C_{51}H_{94}O_7$	+NL 463	832.7
18:0 brassicasteryl	$C_{52}H_{94}O_7$	+NL 463	844.7
18:0 campesteryl	$C_{52}H_{96}O_7$	+NL 463	846.7
18:0 stigmasteryl	$C_{53}H_{96}O_7$	+NL 463	858.7
18:0 sitosteryl	$C_{53}H_{98}O_7$	+NL 463	860.7
20:1 cholesteryl	$C_{53}H_{98}O_7$	+NL 489	860.7
20:1 brassicasteryl	$C_{54}H_{98}O_7$	+NL 489	872.7
20:1 campesteryl	$C_{54}H_{100}O_7$	+NL 489	874.7
20:1 stigmasteryl	$C_{55}H_{100}O_7$	+NL 489	886.7
20:1 sitosteryl	$C_{55}H_{102}O_7$	+NL 489	888.8

Table S3. Mass spectral scan values for steryl glucuronides and rhamnosides.

Class	Formula [M]	Scan mode	Mass of intact ion analyzed $[M+NH_4]^+$ (*m/z*)
**Steryl Glucuronide**			
cholesteryl	$C_{33}H_{54}O_7$	+NL 211	580.4
ergosteryl	$C_{34}H_{52}O_7$	+NL 211	590.4
brassicasteryl	$C_{34}H_{54}O_7$	+NL 211	592.4
campesteryl	$C_{34}H_{56}O_7$	+NL 211	594.4
stigmasteryl	$C_{35}H_{56}O_7$	+NL 211	606.4
sitosteryl	$C_{35}H_{58}O_7$	+NL 211	608.4
**Steryl Rhamnoside**			
cholesteryl	$C_{33}H_{56}O_5$	+NL 181	550.4
ergosteryl	$C_{34}H_{54}O_5$	+NL 181	560.4
brassicasteryl	$C_{34}H_{56}O_5$	+NL 181	562.4
campesteryl	$C_{34}H_{58}O_5$	+NL 181	564.4
stigmasteryl	$C_{35}H_{58}O_5$	+NL 181	576.4
sitosteryl	$C_{35}H_{60}O_5$	+NL 181	578.4

Table S4. List of protein sequences for phylogenetic tree from Fig. 1.
Amino acid sequences are shown in the same order as in Fig. 1, from top to bottom. GenBank accession numbers and amino acid lengths are listed. Asterisks indicate sequences that were not assigned UGT names due to incomplete sequence or lack of EST support.

**Name in Phylogenetic Tree**	**Official Names**	**GenBank Accession**	**Amino Acids**
Arabidopsis thaliana UGT80A2	UGT80A2/At3g07020	NP_566297.2	637
Arabidopsis lyrata UGT80A12	UGT80A12	XP_002884620.1	635
Capsella rubella UGT80A13	UGT80A13	XP_006297184.1	642
Glycine max UGT80A14	UGT80A14	XP_003552079.1	593
Vitis vinifera UGT80A15	UGT80A15	XP_002265023	662
Populus trichocarpa UGT80A16	UGT80A16	XP_002321244.2	619
Solanum lycopersicum UGT80A17	UGT80A17	XP_004240462.1	641
Brachypodium distachyon UGT80A18	UGT80A18	XP_003580955.1	614
Zea mays UGT80A19	UGT80A19	NP_001151796.1	546
Ricinus communis UGT80A21	UGT80A21	XP_002512608.1	597
Physcomitrella patens UGT80A8	UGT80A8	XP_001780556.1	516
Selaginella moellendorffii UGT80A20	UGT80A20	XP_002989345.1	551
Arabidopsis thaliana UGT80B1	UGT80B1/At1g43620	NP_175027.1	615
Arabidopsis lyrata UGT80B5	UGT80B5	XP_002893951.1	625
Capsella rubella UGT80B11	UGT80B11	XP_006307008.1	620
Glycine max UGT80B6	UGT80B6	XP_003535566	625
Vitis vinifera UGT80B7	UGT80B7	XP_002265312.1	682
*Ricinus communis 80B**		XP_002524293.1	644
Populus trichocarpa UGT80B8	UGT80B8	XP_002302172.1	616
Solanum lycopersicum UGT80B9	UGT80B9	XP_004237799.1	643
Brachypodium distachyon UGT80B3	UGT80B3	XP_003564697.1	617
Zea mays UGT80B10	UGT80B10	NP_001132100.1	620
*Aspergillus kiwachii**		GAA88361.1	833
*Aspergillus niger**		XP_001393023.2	880
*Fusarium graminearum**		XP_391364.1	748
*Nectria haematococca**		XP_003053667.1	834
*Botryotinia fuckeliana**		XP_001548189.1	1210
*Schizophyllum commune**		XP_003030067	517
*Serpula lacrymans**		EGN96360.1	563
Arabidopsis thaliana UGT713B1	UGT713B1/At5g24750	NP_568452.2	520
Arabidopsis lyrata UGT713B2	UGT713B2	XP_002874220.1	522
Capsella rubella UGT713B3	UGT713B3	XP_006289138.1	517
Solanum lycopersicum UGT713C3	UGT713C3	XP_004230516.1	506
Vitis vinifera UGT713C1	UGT713C1	XP_002273501.2	599
Ricinus communis UGT713C4	UGT713C4	XP_002528612.1	512
*Populus trichocarpa 713C**		XP_002303168.2	389
Glycine max UGT713C2	UGT713C2	XP_003532979.2	498
Brachypodium distachyon UGT713A1	UGT713A1	XP_003571635.1	522
Zea mays UGT713A2	UGT713A2	NP_001183260.1	532
Physcomitrella patens UGT718A1	UGT718A1	XP_001770940.1	507
Selaginella moellendorffii UGT719A1	UGT719A1	XP_002964753.1	558
Saccharomyces cerevisiae UGT51A1	UGT51/UGT51A1	Q06321	1198
Arabidopsis thaliana GCS	GCS/At2g19880	NP_001189555.1	520

Table S5. SG, ASG, steryl glucuronides, and steryl rhamnosides quantification in *ugt80* and *ugt713* mutant seeds. Mass spectral signals are shown in nmol per mg dry seed weight. Averages are indicated for n=5, with standard deviations in parentheses. Significant decreases from Col wild-type (WT) are indicated by a single asterisk ($P \leq 0.05$) or double asterisk ($P \leq 0.0001$). See Figs. 4 and 5 for graphical representations of the data.

	**WT Col**	***ugt80A2***	***ugt80B1***	***ugt713B1***	***ugt80A2,B1***	***ugt80A2,713B1***	***ugt80B1,713B1***	***ugt80A2,B1,713B1***
**Total SGs**	8.556 (1.020)	1.862** (0.460)	7.854 (0.930)	10.469 (1.036)	0.705** (0.133)	2.179** (0.846)	7.669 (1.274)	0.721** (0.110)
**sitosteryl**	6.289 (0.767)	1.223** (0.306)	6.170 (0.874)	7.785 (0.752)	0.513** (0.099)	1.525** (0.649)	6.018 (1.022)	0.542** (0.090)
**campesteryl**	1.513 (0.174)	0.503** (0.116)	1.078* (0.147)	1.802 (0.235)	0.121** (0.020)	0.483** (0.122)	1.001* (0.143)	0.117** (0.016)
**stigmasteryl**	0.597 (0.068)	0.086** (0.029)	0.563 (0.078)	0.705 (0.041)	0.052** (0.014)	0.130** (0.108)	0.556 (0.117)	0.046** (0.005)
**brassicasteryl**	0.125 (0.021)	0.038* (0.009)	0.081* (0.010)	0.138 (0.010)	0.010* (0.002)	0.031* (0.006)	0.075* (0.009)	0.010* (0.002)
**cholesteryl**	0.032 (0.006)	0.012* (0.003)	0.018* (0.004)	0.038 (0.004)	0.008* (0.003)	0.010* (0.001)	0.019* (0.002)	0.006* (0.002)
**Total ASGs**	5.503 (0.912)	5.070 (0.939)	5.471 (1.304)	7.793 (0.644)	1.159* (0.220)	5.619 (1.546)	5.438 (1.130)	1.343* (0.084)
**Total 16:0**	2.618 (0.525)	2.694 (0.514)	3.157 (0.961)	4.242 (0.514)	0.565* (0.121)	3.143 (0.884)	3.319 (0.697)	0.647* (0.061)
**16:0 sitosteryl**	1.414 (0.322)	1.421 (0.273)	1.877 (0.581)	2.326 (0.303)	0.335* (0.076)	1.702 (0.486)	1.981 (0.410)	0.400* (0.043)
**16:0 campesteryl**	1.098 (0.207)	1.177 (0.224)	1.179 (0.361)	1.781 (0.207)	0.198* (0.040)	1.333 (0.375)	1.240 (0.268)	0.209* (0.020)
**16:0 stigmasteryl**	0.053 (0.012)	0.053 (0.011)	0.062 (0.017)	0.072 (0.009)	0.018* (0.003)	0.061 (0.018)	0.061 (0.010)	0.019* (0.003)
**16:0 brassicasteryl**	0.039 (0.015)	0.033 (0.006)	0.028 (0.007)	0.051 (0.010)	0.007* (0.002)	0.036 (0.009)	0.025 (0.006)	0.007* (0.005)
**16:0 cholesteryl**	0.015 (0.013)	0.010 (0.003)	0.011 (0.004)	0.013 (0.005)	0.008 (0.005)	0.011 (0.001)	0.012 (0.005)	0.011 (0.003)
**Total 18:3**	0.720 (0.114)	0.595 (0.125)	0.388* (0.129)	0.914 (0.065)	0.088* (0.022)	0.615 (0.194)	0.302* (0.085)	0.091* (0.012)
**18:3 sitosteryl**	0.501 (0.107)	0.415 (0.085)	0.289* (0.087)	0.666 (0.046)	0.057* (0.016)	0.433 (0.140)	0.224* (0.070)	0.054* (0.005)
**18:3 campesteryl**	0.158 (0.043)	0.130 (0.026)	0.054* (0.010)	0.184 (0.025)	0.017* (0.003)	0.141 (0.044)	0.047* (0.011)	0.017* (0.001)
**18:3 stigmasteryl**	0.029 (0.007)	0.027 (0.007)	0.016* (0.002)	0.031 (0.002)	0.008* (0.003)	0.025 (0.006)	0.015* (0.003)	0.007* (0.003)
**18:3 brassicasteryl**	0.020 (0.012)	0.015 (0.006)	0.009 (0.004)	0.018 (0.007)	0.003* (0.002)	0.012 (0.003)	0.008 (0.003)	0.004* (0.003)
**18:3 cholesteryl**	0.011 (0.011)	0.007 (0.004)	0.020 (0.032)	0.014 (0.016)	0.004 (0.002)	0.005 (0.004)	0.008 (0.012)	0.008 (0.011)
**Total 18:2**	0.504 (0.085)	0.355* (0.065)	0.542 (0.172)	0.644 (0.057)	0.143* (0.026)	0.449 (0.149)	0.484 (0.110)	0.178* (0.010)
**18:2 sitosteryl**	0.324 (0.055)	0.230* (0.043)	0.365 (0.126)	0.416 (0.031)	0.088* (0.017)	0.297 (0.103)	0.331 (0.067)	0.109* (0.006)
**18:2 campesteryl**	0.114 (0.017)	0.084* (0.016)	0.103 (0.029)	0.164 (0.024)	0.029* (0.006)	0.109 (0.035)	0.097 (0.027)	0.032* (0.005)
**18:2 stigmasteryl**	0.029 (0.010)	0.019 (0.006)	0.032 (0.017)	0.027 (0.006)	0.008* (0.001)	0.022 (0.009)	0.025 (0.006)	0.009* (0.002)
**18:2 brassicasteryl**	0.018 (0.016)	0.009 (0.002)	0.011 (0.008)	0.016 (0.009)	0.004 (0.001)	0.008 (0.003)	0.008 (0.003)	0.005 (0.001)
**18:2 cholesteryl**	0.019 (0.018)	0.013 (0.002)	0.031 (0.029)	0.021 (0.018)	0.016 (0.004)	0.012 (0.004)	0.023 (0.017)	0.023 (0.006)
**Total 18:1**	1.203 (0.195)	1.090 (0.192)	0.923* (0.174)	1.486 (0.138)	0.201* (0.035)	1.059 (0.247)	0.912* (0.192)	0.237* (0.019)
**18:1 sitosteryl**	0.814 (0.153)	0.742 (0.131)	0.602* (0.131)	0.997 (0.057)	0.122* (0.027)	0.728 (0.171)	0.623 (0.118)	0.136* (0.010)
**18:1 campesteryl**	0.278 (0.045)	0.269 (0.048)	0.224 (0.044)	0.369 (0.031)	0.038* (0.007)	0.262 (0.067)	0.214 (0.050)	0.047* (0.007)
**18:1 stigmasteryl**	0.035 (0.009)	0.034 (0.007)	0.029 (0.011)	0.036 (0.007)	0.009* (0.002)	0.029 (0.007)	0.026 (0.004)	0.009* (0.004)
**18:1 brassicasteryl**	0.024 (0.012)	0.021 (0.004)	0.017 (0.011)	0.034 (0.032)	0.005* (0.001)	0.016 (0.002)	0.012 (0.005)	0.006* (0.003)
**18:1 cholesteryl**	0.052 (0.073)	0.024 (0.007)	0.051 (0.045)	0.049 (0.068)	0.027 (0.009)	0.024 (0.007)	0.037 (0.033)	0.038 (0.009)

	**WT Col**	***ugt80A2***	***ugt80B1***	***ugt713B1***	***ugt80A2,B1***	***ugt80A2,713B1***	***ugt80B1,713B1***	***ugt80A2,B1,713B1***
**Total 18:0**	0.267 (0.115)	0.205 (0.039)	0.266 (0.064)	0.311 (0.106)	0.089* (0.013)	0.224 (0.064)	0.238 (0.062)	0.101* (0.007)
**18:0 sitosteryl**	0.122 (0.032)	0.113 (0.019)	0.141 (0.043)	0.152(0.014)	0.036* (0.009)	0.123 (0.038)	0.116 (0.019)	0.040* (0.005)
**18:0 campesteryl**	0.086 (0.061)	0.059 (0.010)	0.075 (0.024)	0.101 (0.043)	0.017 (0.003)	0.063 (0.018)	0.062 (0.014)	0.019 (0.003)
**18:0 stigmasteryl**	0.011 (0.007)	0.007 (0.001)	0.015 (0.007)	0.010 (0.005)	0.006 (0.003)	0.010 (0.004)	0.008 (0.003)	0.005 (0.002)
**18:0 brassicasteryl**	0.016 (0.027)	0.006 (0.002)	0.007 (0.007)	0.020 (0.035)	0.002 (0.001)	0.004 (0.000)	0.004 (0.002)	0.002 (0.002)
**18:0 cholesteryl**	0.032 (0.020)	0.020 (0.009)	0.027 (0.012)	0.027 (0.015)	0.029 (0.008)	0.024 (0.006)	0.048 (0.041)	0.035 (0.005)
**Total 20:1**	0.191 (0.087)	0.131 (0.022)	0.196 (0.042)	0.197 (0.020)	0.072* (0.014)	0.131 (0.022)	0.184 (0.034)	0.101 (0.007)
**20:1 sitosteryl**	0.000 (0.000)	0.000 (0.000)	0.000 (0.000)	0.000 (0.000)	0.000 (0.000)	0.000 (0.000)	0.000 (0.000)	0.000 (0.000)
**20:1 campesteryl**	0.000 (0.000)	0.000 (0.000)	0.000 (0.000)	0.000 (0.000)	0.000 (0.000)	0.000 (0.000)	0.000 (0.000)	0.000 (0.000)
**20:1 stigmasteryl**	0.094 (0.015)	0.075 (0.014)	0.128 (0.034)	0.113 (0.005)	0.034* (0.009)	0.077 (0.014)	0.124 (0.024)	0.045* (0.008)
**20:1 brassicasteryl**	0.097 (0.084)	0.057 (0.010)	0.068 (0.010)	0.084 (0.018)	0.038 (0.007)	0.053 (0.009)	0.060 (0.011)	0.045 (0.006)
**20:1 cholesteryl**	0.000 (0.000)	0.000 (0.000)	0.000 (0.000)	0.000 (0.000)	0.000 (0.000)	0.000 (0.000)	0.000 (0.000)	0.000 (0.000)
**Total Steryl Glucuronides**	0.057 (0.010)	0.053 (0.026)	0.071 (0.028)	0.066 (0.028)	(0.067) (0.017)	0.064 (0.022)	0.074 (0.010)	0.072 (0.022)
**sitosteryl**	0.035 (0.011)	0.035 (0.018)	0.054 (0.022)	0.044 (0.018)	0.046 (0.011)	0.043 (0.013)	0.057 (0.019)	0.050 (0.017)
**campesteryl**	0.010 (0.004)	0.011 (0.005)	0.015 (0.005)	0.014 (0.006)	0.012 (0.004)	0.013 (0.004)	0.015 (0.004)	0.013 (0.003)
**stigmasteryl**	0.004 (0.002)	0.004 (0.002)	0.005 (0.002)	0.004 (0.002)	0.006 (0.002)	0.005 (0.002)	0.005 (0.001)	0.006 (0.002)
**brassicasteryl**	0.002 (0.001)	0.003 (0.002)	0.003 (0.003)	0.004 (0.002)	0.003 (0.002)	0.003 (0.002)	0.004 (0.001)	0.004 (0.001)
**Total Steryl Rhamnosides**	0.017 (0.006)	0.017 (0.011)	0.018 (0.005)	0.023 (0.014)	0.017 (0.008)	0.016 (0.008)	0.018 (0.006)	0.021 (0.010)
**sitosteryl**	0.008 (0.004)	0.009 (0.006)	0.010 (0.003)	0.012 (0.008)	0.010 (0.003)	0.009 (0.003)	0.011 (0.003)	0.013 (0.006)
**campesteryl**	0.004 (0.002)	0.003 (0.002)	0.004 (0.002)	0.004 (0.003)	0.004 (0.002)	0.003 (0.001)	0.003 (0.001)	0.004 (0.001)
**stigmasteryl**	0.003 (0.001)	0.003 (0.002)	0.003 (0.002)	0.004 (0.002)	0.002 (0.001)	0.003 (0.002)	0.003 (0.002)	0.002 (0.001)
**brassicasteryl**	0.002 (0.001)	0.002 (0.001)	0.002 (0.001)	0.002 (0.002)	0.001 (0.002)	0.002 (0.001)	0.003 (0.001)	0.002 (0.001)

Table S6. *In vitro* sterol glucosyltransferase activity of UGT80A2, UGT80B1, and UGT713B1 enyzmes. Enzymes were expressed in yeast (See Materials and Methods). Microsomal extracts were mixed with sterol substrates and either UDP-glucose or UDP-glucuronide. Products of the reactions were analyzed by targeted ESI-MS/MS scans. Averages of normalized signals and standard deviations (parentheses) are shown for n=3 replicates. Significant increases in activity over the control are indicated by asterisk ($P \leq 0.05$). See Fig. 6 for graphical representations of the data for UDP-glucose.

**UDP-Substrate**<br>**Sterol Substrate**	**UDP-Glucose**	**UDP-Glucuronide**
**Cholesterol**		
Control	0.015 (0.001)	0.000 (0.000)
UGT80A2	5.272* (2.861)	0.000 (0.000)
UGT80B1	2.282* (0.249)	0.029 (0.050)
UGT713B1	0.018 (0.011)	0.000 (0.000)
**Ergosterol**		
Control	0.021 (0.010)	0.002 (0.000)
UGT80A2	34.5* (11.9)	0.003 (0.000)
UGT80B1	29.2* (9.6)	0.003 (0.000)
UGT713B1	0.004 (0.003)	0.010 (0.011)
**Sterol mixture (brassica- + campe- + sito- + stigmasterol)**		
**Brassicasterol**		
Control	0.035 (0.010)	0.012 (0.011)
UGT80A2	2.89* (1.23)	0.016 (0.002)
UGT80B1	1.14* (0.40)	0.009 (0.006)
UGT713B1	0.032 (0.000)	0.012 (0.006)
**Campesterol**		
Control	0.012 (0.003)	0.001 (0.001)
UGT80A2	1.97* (0.82)	0.001 (0.001)
UGT80B1	0.217* (0.087)	0.000 (0.000)
UGT713B1	0.015 (0.013)	0.004 (0.002)
**Sitosterol**		
Control	0.043 (0.030)	0.005 (0.001)
UGT80A2	4.24* (1.75)	0.004 (0.000)
UGT80B1	0.290* (0.100)	0.004 (0.001)
UGT713B1	0.034 (0.023)	0.005 (0.001)
**Stigmasterol**		
Control	0.017 (0.004)	0.001 (0.000)
UGT80A2	1.58* (0.71)	0.001 (0.000)
UGT80B1	0.344* (0.142)	0.001 (0.000)
UGT713B1	0.012 (0.011)	0.001 (0.001)
